# Nano-Antagonist Alleviates Inflammation and Allows for MRI of Atherosclerosis

**DOI:** 10.7150/ntno.37391

**Published:** 2019-11-01

**Authors:** Brian Mog, Courteney Asase, Alice Chaplin, Huiyun Gao, Sanjay Rajagopalan, Andrei Maiseyeu

**Affiliations:** 1 Davis Heart and Lung Research Institute and the Division of Cardiovascular Medicine, Department of Internal Medicine, The Ohio State University College of Medicine, Columbus, Ohio, 420 West 12th Avenue, Columbus, OH 43210, USA;; 2 Cardiovascular Research Institute, Case Western Reserve University, School of Medicine, 10900 Euclid Ave, Cleveland, OH 44106, USA.

**Keywords:** Nanoparticles, peptides, CCR2, atherosclerosis, MRI

## Abstract

Specific targeting of inflammation remains a challenge in many pathologies, because of the necessary balance between host tolerance and efficacious inflammation resolution. Here, we discovered a short, 4-mer peptide which possesses antagonist properties towards CC chemokine receptor 2 (CCR2), but only when displayed on the surface of lipid nanoparticles. According to BLAST analysis, this peptide motif is a common repeating fragment in a number of proteins of the CC chemokine family, which are key players in the inflammatory response. In this study, self-assembled, peptide-conjugated nanoparticles (CCTV) exhibited typical properties of CCR2 antagonism, including affinity to the CCR2 receptor, inhibition of chemotactic migration of primary monocytes, and prevention from CC chemokine ligand 2 (CCL2)-induced actin polymerization. Furthermore, CCTV ameliorated NFkB activation and downregulated the secondary, but not the primary, inflammatory response in cultured macrophages. When conjugated with gadolinium or europium cryptates, CCTV enabled targeted imaging (via magnetic resonance imaging and time-resolved fluorescence) of atherosclerosis, a chronic inflammatory condition in which the CCL2/CCR2 axis is highly dysfunctional. CCTV targeted CCR2^hi^Ly6C^hi^ inflammatory monocytes in blood and the atherosclerotic plaque, resulting in cell-specific transcriptional downregulation of key inflammatory genes. Finally, CCTV generated pronounced inflammasome inactivation, likely mediated through reactive oxygen species scavenging and downregulation of NLRP3. In summary, our work demonstrates for the first time that a short peptide fragment presented on a nanoparticle surface exhibit potent receptor-targeted antagonist effects, which are not seen with the peptide alone. Unlike commonly used cargo-carrying, vector-directed drug delivery vehicles, CCTV nanoparticles may act as therapeutics/theranostics themselves, particularly in inflammatory conditions with CCL2/CCR2 pathogenesis, including cardiovascular disease and cancer.

## Introduction

Inflammation is a process of immune system activation that is often accompanied by increased migration of immune cells to a site of injury or disease. Inflammation-driven immune cell migration is orchestrated via chemokine receptor-ligand pairs that are ubiquitously expressed in innate immune cells and are hyperactivated in many pathologies. Interruption of chemokine signaling pathways through various interventions, such as RNAi 1,2, small molecules^3^ or nanotherapeutics 2, has previously been shown to reduce pathologic inflammation in cardiovascular and neurologic disorders, diabetes, and cancer^4^. The challenge, however, remains to restrain immune system hyper-responsiveness without complete cessation of the inflammatory response. Excessive immune activation in disease may lead to irreversible tissue damage - a prime example of which is septic shock, a severe uncontrollable inflammatory reaction to bacterial pathogens with 40-60% mortality rates. Conversely, excessive immune suppression through external anti-inflammatory therapies may compromise the ability of a patient to promptly respond to pathogenic invaders (primary response to inflammation). Therapeutic “over silencing” of the immune response may occur when an anti-inflammatory treatment non-specifically targets molecular cues, such as cytokines, that signal peripheral immune cells to engage in host defense. For instance, in the recent CANTOS^5^ clinical trial that evaluated the anti-inflammatory effects of the interleukin-1 beta (IL-1β) neutralizing monoclonal antibody canakinumab in cardiovascular disease, a reduction in cardiovascular events was observed at the cost of a significantly increased risk of death from infection and sepsis.

The IL-1β pathway is a well-recognized target in many inflammatory diseases, including atherosclerosis, a plaque formation-driven narrowing of blood vessels which can lead to devastating heart attacks and strokes. Therapeutic approaches to target IL-1β in clinical settings are mostly limited to downstream targeting, i.e. systemic neutralization of the IL-1β itself via scavenging antibodies (such as canakinumab). Targeting upstream, i.e. inactivation of specific biological pathways regulating IL-1β production is an alternative strategy that holds significant promise, as it may allow for reduced side-effects while keeping systemic immunity in check. For example, besides fatal sepsis cases observed in CANTOS trial^5^, IL-1β inhibition in systemic juvenile idiopathic arthritis increased risk of urinary tract infections, nasopharyngitis, thrombocytopenia and severe neutropenia^6^,^7^. Therefore, “upstream targeting” can be particularly efficacious if it is accomplished at the site of inflammation, e.g. atherosclerotic plaque. Selective targeting of atherosclerosis via nanotheranostics is being pursued by us and others.^8^-^10^ However many challenges still remain, including complexity of targeted vehicles, which are often equipped with a drug, imaging agent, and a targeting vector - all in the same formulation.

We serendipitously discovered that a short peptide spanning just four amino acids, two cysteines, threonine and valine, reduces inflammation, but only when presented on a nanoparticle surface. Peptide-conjugated lipids were self-assembled into nanoparticles that we call CCTV (one letter symbols of the corresponding peptide sequence). CCTV were found to target CC chemokine receptor 2 (CCR2), which is highly expressed on inflammatory cells and in atherosclerotic plaques. These simple, single-component nanoparticles significantly ameliorated monocyte migration and resolved secondary inflammatory responses, particularly IL-1β, without a demonstrable effect on mediators of the primary immune response. We demonstrate that CCTV target the IL-1β pathway upstream, while its high affinity to CCR2 allowed for atherosclerosis delivery and imaging. Our work proposes CCTV as an attractive candidate for the development of anti-inflammatory therapeutics that mediates efficacious site-specific targeting of CCR2/CCL2 axis without inhibition of the primary inflammatory response characteristic of other inflammation-targeted therapies.

## Materials and Methods

All reagents, their origin, identification and vendor catalogue numbers are presented in **Supplementary Table S1**. This includes cell culture supplies, PCR primers, ELISA and biochemical kits, antibodies and other reagents and resources. Abbreviations can be found in **Supplementary Table S1**.

### Support Vector Machine (SVM) predictions

An *in silico* SVM prediction platform was previously described for small and tiny peptide sequences.^11^ The publicly-available prediction tool (http://crdd.osdd.net/raghava/ahtpin) was used to generate tetrapeptide sequences derived from human CC motif chemokine 2 [UniProtKB - P13500 (CCL2_HUMAN)]. The prediction model used was “amino acid composition” and the SVM threshold was set to -0.8. The top 50 peptides were selected and filtered by SVM score and prediction outcome (AHT were selected and Non-AHT were omitted). See** Supplementary Figure S1 and Supplementary Table S2** for peptides and their characteristics.

### Nanoparticle synthesis

**Figure 1:**
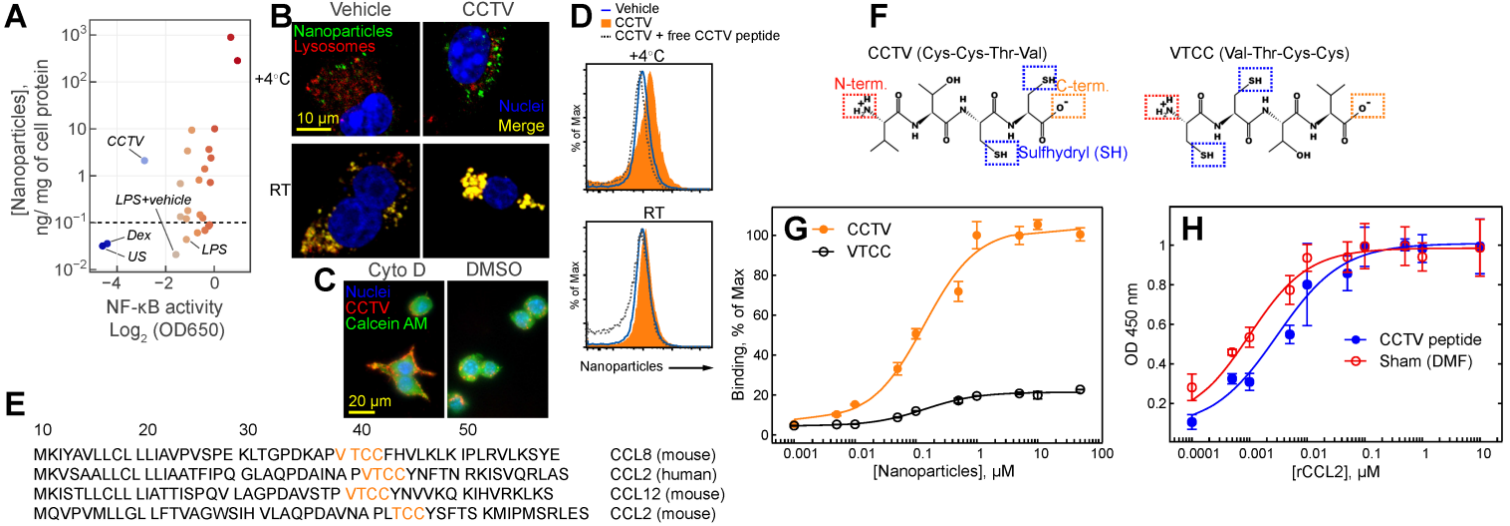
** Screening identifies CCTV as an anti-inflammatory nanoparticle with high affinity for CC chemokine receptor 2. A)** A screening of a small library of nanoparticles in lipopolysaccharide (LPS)-stimulated macrophage reporter cells (RAW-Blue) identified CCTV, which downregulates nuclear factor-kappa B (NF-κB) activity. Each point on the plot represents a relative activity of NF-κB (X-coordinate) and corresponding nanoparticle content per mg of cell protein (Y-coordinate) calculated from nanoparticle fluorescence. Y-axis values <10^-1^ (horizontal dash line) are indicative of autofluorescence measured in the cells and do not represent concentrations. Dexamethasone (Dex) is an NF-κB inhibitor and was used as a positive control. Vehicle-treated and unstained (US) cells served as a negative control. **B)** CCTV binding to the cell surface was validated in RAW cells using fluorescence microscopy. The cells were stained at 4^o^C or room temperature for 30 min with CCTV or vehicle nanoparticles (sudo-colored green). Lysosomes were visualized after staining with LAMP1 antibody (red). Nuclei were stained using Hoechst 33342 (blue). **C)** The uptake of CCTV was inhibited in the presence of 5 µM cytochalasin D (cyto D)**.** DMSO served as vehicle control. Calcein AM was used for whole cell staining.** D)** CCTV binding to a receptor on the cell surface was studied by flow cytometry in the presence and absence of the excess (1 mM) of the free peptide. **E)** The Basic Local Alignment Search Tool (BLAST) analysis determined that CCTV peptide sequence repeats in mouse and human CC chemokine receptor family. Only the first 50 amino acids are shown. **F)** Chemical structure of CCTV and its mirror analog VTCC and their possible conjugation strategies that include N- (N-term.) and C-terminus (C-term.) as well as sulfhydryl attachment. **G)** CCTV displayed affinity to CC chemokine receptor 2 (CCR2) and bound to immobilized recombinant receptor protein in a dose dependent manner. **H)** Excess of the free CCTV peptide (1 mM) insignificantly competed with receptor binding of natural CCR2 ligand - CCL2. DMF = dimethylformamide, was used to solubilize free CCTV peptide. Error bars are standard deviation of three independent experiments.

Lipid-peptide conjugates were synthesized from either DSPE-PEG-CO_2_H or DSPE-PEG-MAL using peptides derived using SVM prediction platform and listed in **Supplementary Table S2**. The peptides were conjugated to either the N-terminus or via sulfhydryl groups as depicted in **Figure 1E**. Lipid films from both PEGylated derivatives were prepared by evaporation under nitrogen gas, from 1 µM of lipid solution in chloroform. Tetrapeptides were custom-synthesized by Genscript. 5 µmol of peptide solutions in water:dimethylformamide (1:1) were prepared via a brief bath sonication. Some peptides did not completely dissolve, resulting in a suspension. PEGylated lipids prepared above were sonicated (10 min, 300 W, room temperature) in 5 mL of MES (pH 6, 100 mM) for DSPE-PEG-CO_2_H and in 5 mL of HEPES buffered saline [HBS] (pH 7.2, 100 mM, 154 mM NaCl) for DSPE-PEG-MAL. Next, DSPE-PEG-CO_2_H was activated by the addition of 2 mg of EDC (20 mg/mL in water) and 5 mg of sulfo-NHS (10 mg/mL in water). This was sonicated at 15 W for 10 min in a temperature controlled bath at 30^o^C. Then, peptide solutions/suspensions were added to the resulting DSPE-PEG-NHS or previously prepared DSPE-PEG-MAL and stirred at room temperature for 4 h. The reaction with DSPE-PEG-NHS was quenched by the addition of 100 µL of 0.5 M of NH_2_OH in HBS, plus 1 mM EDTA. The reaction with DSPE-PEG-MAL was quenched by the addition of 7 µL of 2-mercaptoethanol. The solution was freezed at -80^o^C and lyophilized. Next, lipid-peptide conjugates were extracted with chloroform (3 x 2 mL) and centrifuged at 4000 g x 5 min. Combined extract supernatants were evaporated under N_2_ gas and sonicated (10 min, 100 W) in 0.5-1 mL of water at room temperature. This raw product was purified by size extrusion chromatography as follows. Millipore Vantage column (10 x 500 mm) was packed with Sepharose 4B in deionized water under flow rate of 0.5 mL/min. Next, 0.5 mL of raw nanoparticle preparation was injected into the column and 0.5 mL fractions were collected at 0.5 mL/min simultaneously with UV detection at 190, 214, 250 and 280 nm using the Bio Rad DuoFlow system equipped with BioLogic QuadTec UV/Vis detector. See **Supplementary Figure S2** for representative chromatograms. Combined fractions 15-31 were frozen and lyophilized. Particles used in signalling *In vitro* work were prepared by reconstitution of lyophilizates in PBS or HBS buffer via sonication. Particles used in reporter, flow cytometry and microscopy experiments were prepared by addition of 1% (by weight) of Rhodamine-PE in ethanol followed by reconstitution as described above. Particles used in magnetic resonance imaging (MRI) and time-resolved fluorescence (TRF) assays were prepared by addition of 10-20% (by weight) of gadolinium or europium chelate-lipids followed by reconstitution as above. Gadolinium-DTPA (18:0 PE-DTPA) was obtained from Avanti Polar Lipids and europium cryptate-NHS was obtained from Aat Bioquest (**Supplementary Table S1**). Europium cryptate-NHS was further conjugated to phosphatidylethanolamine (PE) to obtain Eu-cryptate-PE according to published and well established protocols 12. To confirm peptide conjugation, the powder was reconstituted in 1 mL of chloroform and analyzed with TLC (eluent chloroform:methanol:water 65:25:4) detecting with Molybdenum blue (**Supplementary Figure S3A**) or Dragendorff reagent (**Supplementary Figure S3B**). Nanoparticles were characterized by dynamic light scattering using Malvern Zetasizer Nano S (**Supplementary Table S3**). The presence of the peptide bond (N-H) and aliphatic C-H and C-C stretches (lipids) was confirmed by infrared (IR) spectroscopy. Nanoparticles were isolated as a homogeneous population (peak) in gel-filtration (see above) and were used without additional purification.

### Nanoparticle screening: NFkB activation and cellular uptake

A schematic representation of nanoparticle screening is depicted in **Supplementary Figure S4**. Briefly, RAW-Blue *NFkB* reporter cells were cultured as per the manufacturer's recommendations. Cells were seeded and cultured overnight in black opaque 96-well plates with a clear bottom at 50,000 per well. Cells were then treated with 1 and 10 µM of nanoparticles containing 1% rhodamine and positive and negative controls (see below) for 30 min on ice followed by washing steps with cold PBS. Next, lipopolysaccharide (LPS) was added at 100 ng/mL and incubated for 6 h at 37^o^C, following which the cells were washed again and incubated for 18 h at 37^o^C in fresh medium. Next, 20 µL of supernatant was collected and assayed using SEAP detection reagent in a separate clear bottom 96-well plate as per Invivogen instructions (http://www.invivogen.com). Absorbance at OD 650 nm was recorded and was indicative of NFkB activation. In parallel, cells in the opaque plate were washed again and fluorescence was read at 550 nm excitation and 620 nm emission. This signal from rhodamine was indicative of nanoparticle uptake. Controls were also added to the same plate and consisted of dexamethasone (Dex, 1 mM), N-acetylcysteine (NAC, 10 mM), and vehicle (DSPE-PEG lipids with rhodamine at 10 µM). One set of cells was left completely untreated (no LPS, unstimulated). All analysis was performed using Molecular Devices i3 multi-mode plate reader and SoftMax Pro software version 6.2 (Molecular Devices).

### BLAST analysis

Peptide sequences were aligned using the protein BLAST suite (https://blast.ncbi.nlm.nih.gov). The search set was as follows: Database - Protein Data Bank proteins (pdb), Organism - Homo sapiens, Mus musculus; no exclusions were made. The algorithm was bastp (protein-protein BLAST). Max target sequences were set at 50, threshold was 10 and word size was 2. Sorting parameters included matrix BLOSUM90, gap costs were Existence 10, and composition-based statistics was utilized. Low complexity regions filter was applied.

### CCR2 receptor binding studies

A sandwich-type plate-based immunoassay was developed in-house to study CCR2 receptor binding. Black opaque plates (for fluorescence studies) or clear high protein binding plates (for absorbance studies) were coated with recombinant CCR2 protein at 2 µg/mL in PBS overnight at 4^o^C. Plates were washed 5 times with PBS and treated as follows: for nanoparticles binding, varying concentrations of fluorescent nanoparticles or vehicle control (see above) in PBS were added to the opaque plates and incubated for 4 h at room temperature. For peptide competition/binding studies, recombinant human CCL2 was added in clear plates at varying concentrations in PBS simultaneously with a large excess of peptides (1 mM final concentration, dissolved in dimethylformamide) and incubated for 1 h at room temperature. All plates were washed 5 times with PBS and fluorescence in opaque plates was read at 550 nm excitation and 620 nm emissions. Clear plates were additionally blocked in 5% fish serum in PBS (Aqua Block), washed again and incubated with anti-MCP-1 (anti-CCL2) antibody conjugated to biotin. Clear plates were washed and incubated with 0.1 µg/mL streptavidin-HRP. Finally, clear plates were washed and incubated with SigmaFast OPD reagent in the presence of hydrogen peroxide-urea, as per the manufacturer's instructions. After 30 min incubation period at 37^o^C, the plates were read at 450 nm on a multiwell plate reader as described above. Fluorescence emission values and OD at 450 nm were plotted against concentration using a 4-parametric fit.

### Cell culture

RAW-Blue (Invivogen), RAW cells and J774 cells (ATCC) were cultured as per vendor recommendations. Bone marrow derived macrophages (BMDM) were obtained as previously described by us and others 8,13.

### Immunoblot assays (Western blot)

Immunoblotting was performed as previously described 14 with the following modifications. Briefly, cell lysates were obtained in SDS lysis buffer consisting of 2% SDS, 50 mM Tris-HCl (pH 6.8), 100 mM DTT, 5% Ficoll 400, 0.0001% bromophenol blue and a cocktail of protease inhibitors. Lysates were sonicated on ice before heating at 97^o^C for 7 min to completely denature the protein. Protein concentration was determined using IR spectroscopy (which is insensitive to high SDS and DTT concentrations) with a Millipore DirectDetect spectrometer. Lysates were equally loaded at 10-15 µg per lane on BioRad TGX Criterion gels (4-15%) and resolved at 300 V in Tris-Glycine running buffer. Proteins were transferred on nitrocellulose membranes using the BioRad TransBlot turbo according to the manufacturer's instructions. Membranes were blocked in fish serum (Aqua Block) or 5% nonfat dry milk in TBS-T (0.05% Tween 20) and probed overnight at 4^o^C with primary antibodies as indicated in **Supplementary Table S1.** The membranes were washed in TBS-T and incubated with secondary antibodies for 1 h at room temperature. After washing, semi-quantitative visualization of the proteins of interest was enabled using an enhanced chemiluminescence reagent as previously described 15 and the Azure C400 western blot imaging system.

### Gene expression analysis

RAW 264.7 cells were seeded in 24-well plates at 1.0 x 10^5^ per well with LPS (100 ng/mL) and allowed to attach overnight. Then, cells were treated for 24 h with various nanoparticle formulations as indicated in the figure and figure legend. After a quick wash with PBS, RNA was isolated from cells following the Trizol method. Next, RNA was reverse transcribed to cDNA, and amplified by the polymerase chain reaction (PCR) using reagents from cDNA synthesis kit, SYBR Green PCR Master Mix and target-specific TaqMan probes as indicated in **Supplementary Table S1.** Roche LightCycler 480 II was used to perform quantification analysis of gene expression using the ΔΔCT method as previously described 9,13,16.

Gene expression in atherosclerotic plaque-derived leukocytes was analyzed using Nanostring nCounter assays (mouse inflammation panel). To prepare RNA for this analysis, abdominal aortas from chow-fed (normal) and western diet-fed atherosclerotic mice (see Animals section below) were digested in an enzyme cocktail consisting of liberase (4U/mL), DNAse I (0.1mg/mL), and hyaluronidase (60U/mL) in RPMI-1640 at 37^o^C for 40 min with shaking, followed by separation of leukocytes via density gradient centrifugation in Lympholyte-M (as per manufacturer's instructions) and, finally, RNA isolation from RLT buffer lysates using Qiagen RNA-Easy kit as per manufacturer's instructions. RNA samples were frozen in RNAse-free water and sent to NanoString Technologies (Seattle, WA) in dry ice.

### Cytokine analysis

Cytokines were analyzed by EVE Technologies (https://www.evetechnologies.com) using the Luminex 31-Plex kit. Cell culture supernatants were diluted twice with PBS and provided in biological triplicates to EVE Technologies via overnight shipment in dry ice. Individual IL-1β and TNFɑ assays were performed using Perkin Elmer AlphaLISA kits, as per **Supplementary Table S1** and vendor instructions.

### Chemotaxis assays

**Figure 2:**
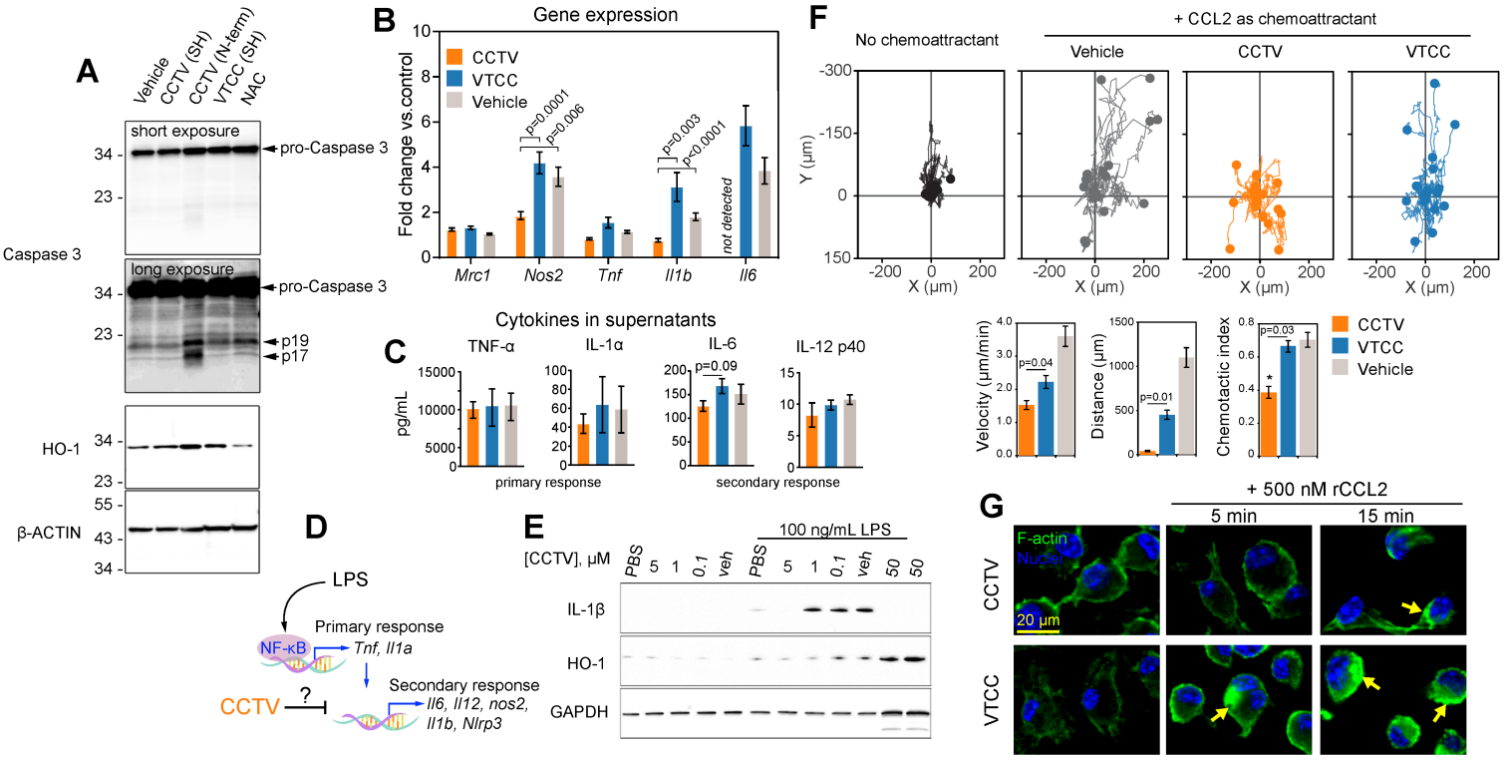
** CCTV downregulates inflammation on a transcriptional and protein level and exhibits prototypical CCR2 antagonism. A)** Depending on the conjugation strategy, CCTV or VTCC demonstrate upregulation of active (cleaved at Asp175, fragments p19/17) caspase 3 and heme oxygenase (HO-1) proteins in cells treated with nanoparticles (1 µM for 3 h) conjugated via N-terminus (N-term), but not through sulfhydryl (SH). N-acetylcysteine (NAC) served as an antioxidant control. β-Actin was used as a loading control. **B)** Gene expression analysis in RAW cells treated with 1 µM of vehicle, CCTV and VTCC (peptide nanoparticle control) for 24 h. mRNA expression of mannose receptor (*Mrc1*), nitric oxide synthase 2 (*Nos2*), tumor necrosis factor (*Tnf*), interleukin-1beta (*Il1b*), and interleukin-6 (*Il6*) were analyzed. **C)** Cytokines in cell culture supernatants were analyzed as indicated. **D)** Schematic representation of transcriptional activation of primary and secondary inflammatory response to lipopolysaccharide (LPS). **E)** Immunoblot analysis of dose-dependent suppression of IL-1β and HO-1 production by CCTV with and without stimulation by 100 ng/mL LPS for 16 h in bone marrow derived macrophages (BMDMs). GAPDH served as a loading control. **F)** Chemotaxis assays in bone marrow derived monocytes treated with 5 µM vehicle, CCTV, VTCC or left untreated. Chemotaxis was stimulated by introducing a gradient of recombinant mouse CCL2. Forty (out of 150-300) cell migration tracks are presented for simplicity. Metrics of chemotactic behaviour i.e. velocity, distance travelled, and chemotactic index were quantified as presented in bar graphs. **G)** BMDMs were pre-treated for 6 h with 5 µM CCTV or VTCC followed by 500 nM CCL2 for the indicated times. Actin polymerization was visualized by phalloidin staining (green “islands” of aggregated actin, arrows). Nuclei were stained using Hoechst 33342 (blue). P values are calculated by t-test and error bars are standard errors of three independent experiments.

Chemotaxis assays were performed using IBIDI µ-Slides as per the manufacturer's instructions and the recently published guide detailing the protocol 17. In brief, BMDMs (3 days in culture after isolation) were plated into µ-slide channels simultaneously with 10 µM nanoparticles or controls as indicated in** Figure 2F**. Cells were allowed to attach for 3 h at 37^o^C, washed with 0.2 mL warm cell culture medium using a pipette tip inserted into µ-channel port, following which 100 nM recombinant mouse CCL2 protein was added as a chemoattractant (or omitted in control slides) to the one of side reservoirs. Cell movement was recorded for a duration of 12 h via phase video-microscopy in a stage-top humidified incubator at 37^o^C and 5% CO_2_ atmosphere. Analysis was performed using the ImageJ IBIDI tracking tool, available free of charge at https://ibidi.com.

### Actin polymerization

BMDMs were seeded and cultured on #1 glass coverslips in the presence or absence of 10 μM CCTV or VTCC for 6 h. Next, cells were washed with PBS and recombinant mouse CCL2 was added to a final concentration of 500 nM. Then, cells were incubated for varying times (indicated in **Figure 2G**), after which they were immediately placed on ice and washed 3 times with cold PBS. Actin polymerization was then visualized following staining with phalloidin Alexa Fluor 633, according to the manufacturer's instructions.

### Animals

Male, ApoE^-/-^ mice (5 weeks of age) were purchased from The Jackson Laboratory and kept in AAALAC-accredited facilities at Case Western Reserve University. The experimental procedures here described were approved by The Institutional Animal Care and Use Committee (IACUC). Animals were housed five per cage and allowed to acclimate in the facility for one week. Throughout the experiment, animals were kept on a 12:12 h light-dark cycle at 22 ^o^C, and both diet and water were provided *ad libitum*. After an initial acclimatization period of 2-3 weeks, the chow diet was switched to a high fat, 1.3% cholesterol diet. Animals continued on this diet for at least 20 weeks, and were then used for the imaging experiments described below.

### Time-resolved fluorescence imaging of excised organs

**Figure 3:**
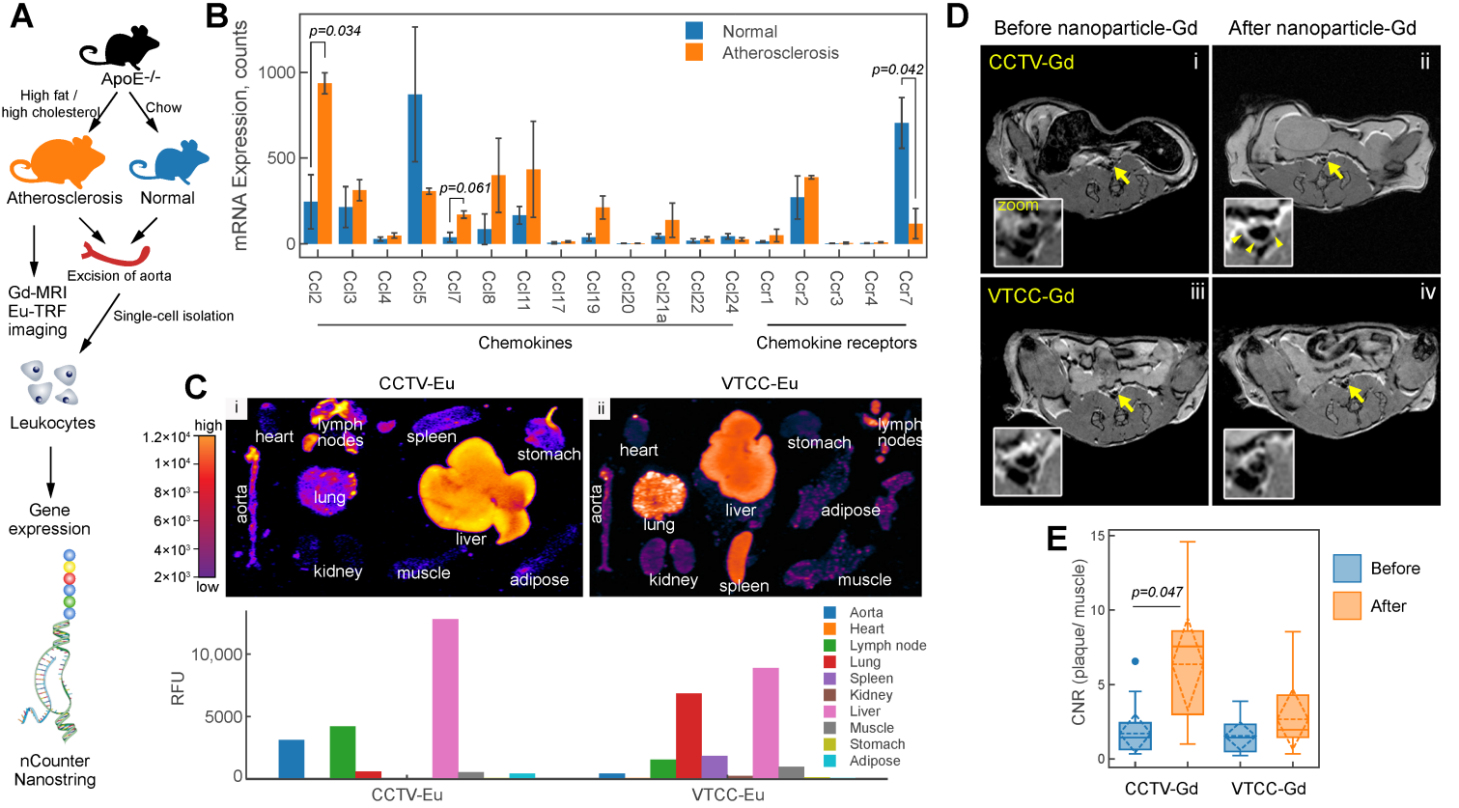
** CCR2/CCL2 is highly expressed in atherosclerosis and a target for CCTV contrast agents. A)** Experimental workflow and generation of atherosclerosis model. **B)** nCounter Nanostring assays enabled quantification of mRNA expression of major chemokines and their receptors in leukocytes isolated from aortas of normal and atherosclerotic mice. **C)** Europium (Eu)-enhanced time resolved fluorescence imaging (TRF) of excised organs from atherosclerotic mice injected with 0.1 mg/kg (based on Eu concentration) of CCTV or VTCC containing 10% (by weight) Eu cryptate-conjugated lipids. Bar graph at the bottom is a quantification of the TRF signal and is representative of the average of two experiments in four mice. **D)** Magnetic resonance imaging of atherosclerotic plaque (arrows) with CCTV and VTCC containing gadolinium (Gd) lipid-conjugated chelates (DOTA) at 20% (by weight) and injected at 0.1 mg/kg (based on Gd concentration). Representative T1-weighted axial images are presented before and 24 h after nanoparticle administration in four mice per group. The inset is a zoomed-in area of the Gd-enhanced abdominal aortic plaque (arrowheads). **E)** Contrast-to-noise ratio (CNR) quantification of gadolinium enhanced plaque. Aortic wall and muscle signals were used to calculate the CNR. Repeated measurements ANOVA followed by pairwise comparisons were performed to analyze the differences in enhancement before vs. after nanoparticle administration.

Two weeks before the experiment, mice were switched to a low-fluorescence, alfalfa-free diet, provided *ad libitum*. Mice were fasted overnight (12-14 h) and then injected with 100 mg/kg of nanoparticles through a penile vein. Twenty four hours later, mice were euthanized and organs were extracted (depicted in **Figure 3**). The organs were positioned on a low-fluorescence mat that was placed on a 96-well plate insert of Molecular Devices i3 plate reader with a TRF module (WB cartridge) and the plate was imaged using excitation of 340 nm and emission 615 nm. Images were analyzed using Molecular Devices SoftMax Pro 6.2 software.

### Magnetic resonance imaging

Imaging of atherosclerosis was performed as previously described by us 8,9. Briefly, utilizing 7T small bore animal imaging magnet Bruker BioSpec, four mice per group were imaged pre and post nanoparticle administration using gradient echo sequences (GRE). Mice were injected with 100 μL of nanoparticle agent at a dose of 0.1 mg/kg (based on gadolinium concentration) and 24 h later placed in the magnet of an MRI scanner for the post contrast scans. Following sequences were used: a) Twelve 0.1x0.1x0.5 mm^3^ transversal contiguous slices, TR/TE=316/3.7 ms, flip angle=20, four averages; b) GRE with pulse sequence parameters were used as follows: RE (repetition time) = 28 milliseconds, ET (echo time) = 4.9 to 10 milliseconds, (FA) flip angle = 30 degrees, 4 averages, resolution = 0.095 mm^2^. 24 slices were acquired covering renal artery to bifurcation. Images were analyzed by OsiriX software available free of charge at www.osirix-viewer.com. Contrast-to-noise ratio (CNR) was calculated as CNR=[signal intensity(plaque) - signal intensity(muscle)]/standard deviation(noise).

### Pharmacokinetic measurements

**Figure 4:**
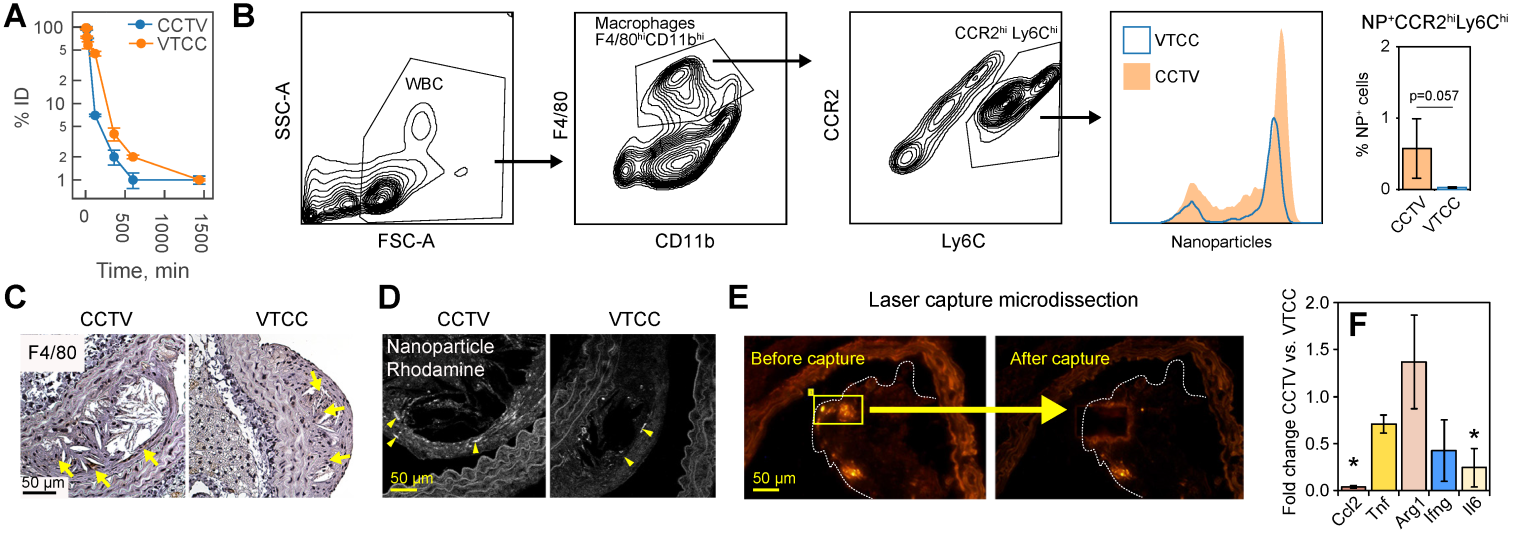
** CCTV targets inflammatory cells expressing CCR2 in blood and atherosclerotic plaque. A)** Blood clearance of CCTV and VTCC over a period of 24 h post injection of 100 mg/kg of nanoparticles in C57BL/6 mice shown as percent of injected dose (% ID). **B)** Blood immune cells are targeted by CCTV in a CCR2-dependent manner. Flow cytometry gating strategy identified inflammatory CCR-positive (CCR2^hi^Ly6C^hi^) cells and percent of these cells positive for nanoparticles (histogram and bar graph). Representative of experiments in three ApoE^-/-^ mice/group injected with 100 mg/kg of nanoparticles. **C)** Immunohistochemistry of atherosclerotic plaque indicating high levels of macrophage-specific marker F4/80. **D)** Rhodamine signal in CCTV but not in VTCC co-stained with respective F4/80 labeling in (C) suggesting CCTV accumulation in plaque macrophages. **E)** Laser capture microdissection (LCM) experiments allowed for isolation of nanoparticle-positive cells in atherosclerotic plaque by means of their rhodamine fluorescence. Representative images are shown for captured areas of nanoparticle-positive cells from the plaque. **F)** mRNA analysis from LCM-captured areas enabled quantification of inflammation-related mRNAs in nanoparticle-targeted cells, including CC chemokine ligand 2 (*Ccl2*), tumor necrosis factor (*Tnf*), arginase 1 (*Arg1*), interferon gamma (*Ifng*) and interleukin 6 (*Il6*). Data are presented as fold change expression in CCTV vs. VTCC. *p<0.05 and representative of 3-4 different animals.

Mice were injected with 100 mg/kg of nanoparticles containing rhodamine (see above) via penile vein and 10-20 μL of blood was drawn from the tail nick into EDTA-coated capillaries at the times indicated in **Figure 4A**. The fluorescence in 4 μL of blood was determined using a Molecular Devices i3 microdrop plate and fluorescence excitation/emission for rhodamine as described above.

### Flow cytometry

Twenty-four hours after nanoparticle injection (see above), mice were euthanized and whole blood was collected via cardiac puncture. The cells were separated from plasma via centrifugation at 2500 rpm for 25 min at 4^o^C and white blood cells were isolated after lysis of red blood cells with ACK lysis buffer. The cells were resuspended in 1% FBS in PBS (FACS buffer) on ice and stained with fluorophore-conjugated antibodies detecting CD11b, CD11c, CCR2, and Ly6C (**Supplementary Table S1**) for 30 min at room temperature. Cells were washed with FACS buffer three times and one time with PBS, and then fixed in 2% paraformaldehyde in PBS, pH 7.4. Samples were run on a Becton Dickinson LSR II flow cytometer and cell surface expression analysis of indicated markers was performed using FlowJo software.

### Immunohistochemistry and laser capture

Twenty-four hours after nanoparticle injection (see above), mice were euthanized and abdominal aortas were isolated and embedded in Optimal Cutting Temperature compound. After cryotome-sectioning, resulting in 20-30 slides per mouse, 15 slides were sent to Ohio State laser capture microdissection core lab for capture and analysis. The rest of the slides were processed as follows: after fixation in methanol:acetone 50:50 at -20^o^C, slides were hydrated in PBS, blocked for 30 min in fish serum (Aqua Block) and stained for 1 h with primary antibodies against F4/80 followed washing and staining with HRP-conjugated secondary antibody for 30 min. DAB peroxidase kit was then used as per manufacturer's instructions followed by mounting. DAB and antibody staining (but not wash steps) was omitted in slides designated for nanoparticle fluorescence visualization. Nanoparticles fluorescence was visualized with Zeiss 510 LSM-meta confocal microscope and 20x objective. DAB F4/80 staining was visualized with Nikon FN1 microscope and same power objective.

### Inflammasome assays

BMDMs or J774 cells were plated in 48-well plates at 2 x 10^5^ per well. Next day, the medium was changed to Opti-MEM (100 μL per well) without serum and the cells were allowed to equilibrate for 3 h. Next, the cells were pretreated with the indicated concentrations of nanoparticles or controls for 3 h followed by the addition of LPS to some wells to the final LPS concentration of 50 ng/mL. LPS priming continued for another 2 h, following which DMSO (vehicle), Nigericin (5 μM) or adenosine triphosphate (ATP) (1 mM) were added to some wells. Cells were incubated for an additional 30 min and cell culture supernatants were collected on ice. Cells were washed with cold PBS once and lysed/processed as described in immunoblotting section. The supernatants (90 μL) were mixed with 10 μL of 10x SDS loading buffer (10% SDS, 10% Ficoll 400, 0.5 M Tris pH 6.8, 0.5 M DTT, and 0.001% bromophenol blue) and heat-denatured as described in the immunoblotting section. Supernatants were loaded at 45 μL per lane on Bio Rad TGX Criterion 12+2 gels (4-20%) and lysates were loaded at 15 μg per lane. Immunoblotting was performed as described above.

### Biochemical assays

GSH /GSSG, lactate dehydrogenase (LDH) and DCFDA assays were performed according to the manufacturer's instructions. The assays were purchased from vendors as indicated in **Supplemental Table S1.**

### Statistical Analysis

Differences between vehicle control and CCTV and VTCC data is presented as mean ± standard error and were analyzed using an independent *t*-test, unless stated otherwise. Statistical significance was assumed at *p*<0.05. Aabel NG software (Gigawiz Ltd. Co) was used for statistical analyses and data representation.

## Results and Discussion

### Screening identifies CCTV nanoparticles with anti-inflammatory potency and affinity to CCR2

A set of 4-mer peptides (tetrapeptides) predicted to have biological activity was derived from a publicly-available peptide predictor tool, utilizing supervised machine learning SVM as previously described 11. The results of this prediction are listed in **Supplementary Table S2** and the physicochemical properties of the obtained peptides are shown in **Supplementary Figure S1.** We used peptides with highest SVM scores because previous study identified high positive correlation coefficients between SVM scores and predicted biological activity 11. Selected peptides with the highest SVM score were synthesized and used further in conjugation to a distal end of polyethylene glycol (PEG)-tagged phospholipids. We synthesized a small library of 18 peptide-lipid conjugates through a carbodiimide chemistry (N-terminus conjugation) or via sulfhydryl-maleimide bond (in peptides with cysteine amino acids). The resulting conjugates were self-assembled into nanoparticles and purified from unreacted peptides by gel-filtration chromatography (**Supplementary Figure S2).** The nanoparticles had a hydrodynamic diameter ranging from 53 to 223 nm and polydispersity indices below 0.2 (**Supplementary Table S3**).

For a targeted nanotherapy to be effective, the nanoparticles must have affinity to a specific cell type, usually determined by cell surface binding. In addition, the nanoparticles themselves or the cargo they carry should alter the phenotype of the targeted cell. For example, inflammation-targeted drug delivery systems are often designed to “switch” macrophage phenotype from “M1-like” to “M2-like”.^18^,^19^ Macrophages are immune cells that act as crucial regulators of the inflammatory immune response that is dysregulated in many diseases. We set out to test whether the synthesized nanoparticles have binding affinity to the macrophage cell surface concomitant with resolving inflammation. Thus, we used the mouse macrophage reporter cell line RAW-Blue that allowed for monitoring activity of Nuclear Factor-kappa B (NF-kB) and also enabled *In vitro* quantitation of nanoparticle binding to the cell surface. NF-kB is a “master regulator” of inflammatory response that controls DNA transcription resulting in production of stress-signaling molecules including inflammatory cytokines. A multistep screening strategy was developed to test both nanoparticle surfaces binding simultaneous with monitoring NF-kB activity (**Supplementary Figure S4**). RAW-Blue cells were pre-treated with two different doses of nanoparticles followed by the addition of lipopolysaccharide (LPS), stimulating the inflammatory response. Most nanoparticles tested had either no effect on NF-kB or further exacerbated inflammation, resulting in an elevated NF-kB activity (**Figure 1A**). One formulation, CCTV, significantly downregulated NF-kB activity while demonstrating high cell-surface binding. Ten micromolar CCTV downregulated NF-kB activities almost to the same level as that in cells treated with NF-kB inhibitor dexamethasone (Dex) at 1 mM. Fluorescence microscopy experiments (**Figure 1B**) conducted at 4^o^C and room temperature in RAW cells confirmed CCTV uptake was receptor-dependent, i.e. CCTV rapidly bound to the cell surface at 4^o^C as compared to the vehicle (phospholipid-PEG nanoparticles without peptide conjugation). On the contrary, uptake of both CCTV and vehicle at room temperature was mediated by endocytosis, as indicated by co-staining of nanoparticle fluorescence with lysosomal protein LAMP1. This was further confirmed by blocking endocytosis with Cytochalasin D followed by incubation with CCTV (**Figure 1C**). Competition experiments performed in the presence of high molar excess of free CCTV peptide did not show appreciable reduction in CCTV staining in RAW cells (**Figure 1D**), suggesting that nanoparticle-CCTV is necessary for binding. BLAST FASTA alignment analysis revealed that CCTV amino acid motif repeats in multiple human and mouse chemokine receptor ligands (**Figure 1E**), as well as in other inflammation-related proteins (**Supplementary Table S4, Supplementary Figure S5**). Interestingly, CCTV aligned with putative receptor binding sites in CCL12 and CCL8 chemokines. This is suggestive that the affinity of CCTV for the cell surface in inflammatory macrophages is mediated by a multivalent interaction with multiple receptors. Because CCL2 (also known as MCP-1) alignment was predicted for both human and mouse chemokines, we further investigated the effects of CCTV on CCL2-CCR2 axis. CCTV that targets multi species CCR2-driven inflammation is advantageous for future research and development as well as possible translation, because this will allow for straightforward testing in mouse models while being predictive of the outcome in humans.

It was previously shown that the truncated form of CCL2 exhibits CCR2 antagonist activity; however, this is strongly dependent on the availability of the N-terminus of the peptide. Thus, acetylated CCL2 peptide analogs displayed significantly reduced binding and activity 20. Considering these prior findings, we synthesized a CCTV mirror-analog nanoparticles, VTCC (**Figure 1F**), that we hypothesized would have diminished CCR2 binding. Indeed, receptor binding assays performed with immobilized recombinant CCR2 demonstrated significant, dose-dependent binding of CCTV but not VTCC nanoparticles (**Figure 1G**). However, in competitive binding assays, excess of free peptide was not able to prevent binding of a natural ligand, CCL2, confirming that nanoparticle architecture is required for receptor binding of CCTV nanoparticles (**Figure 1H**). Additionally, we performed CCTV binding experiments in macrophages derived from CCR2 knockout mice and in wild-type macrophages that were pre-treated with potent CCR2 inhibitor BMS CCR2 22. The uptake of CCTV was drastically reduced in both of these cases (**Supplementary Figure S6**).

### CCTV downregulates inflammation and displays the properties of a CCR2 antagonist

We unexpectedly found that RAW cells incubated with N-terminus-conjugated CCTV (or VTCC, not shown) underwent significant apoptosis and cell death. In fact, cells treated with 1 µM of N-term CCTV or VTCC for just 3 h had less than 50% viability as per MTT assay (data not shown). This was concomitant with significant apoptosis-associated caspase-3 activity, resulting in the formation of the mature active caspases-3, i.e. 17 and 19 kDa fragments (**Figure 2A**). The levels of cellular stress-related heme oxygenase-1 (HO-1) were also increased. However, conjugation through sulfhydryl (SH) negated this effect. It is possible that the presence of two free sulfhydryl groups in CCTV results in endoplasmic reticulum (ER) stress and activation of “initiator” caspases, similar to that in dithiothreitol (which contains two SH groups)-induced apoptosis^21^,^22^. CCTV conjugated via SH possesses only one free SH and were found to have very low toxicity (see below). These findings are potentially useful for cancer targeting, because many tumors overexpress chemokine receptors, particularly CCR2 23,24, and therefore may be targeted via the N-term CCTV. Given these findings, we used only SH-conjugated CCTV and VTCC throughout the rest of our study.

Consistent with the observation that NF-kB activity was inhibited by CCTV, differential gene expression analysis demonstrated that nitric oxide synthase 2 (*Nos2*), interleukin-1 beta (*Il1b*) and interleukin-6 (*Il6*) were significantly downregulated in CCTV-treated, LPS-stimulated RAW cells, as compared to VTCC or vehicle control. This was in contrast to the expression of tumor necrosis factor (*Tnf*), which was unaffected. Nevertheless, a Luminex “inflammation array” panel quantifying levels of cytokines in the cell culture supernatants did not detect significant differences among 14 different cytokines in these treatment groups (**Figure 2C, Supplementary Figure S7**).

LPS-stimulated gene expression is distinct in activation kinetics and can be classified as a primary and secondary transcriptional response (**Figure 2D**). We observed that CCTV affected secondary response genes such as *Il1b* and *Il6*, but not the primary response genes such as *Tnf^25^,^26^*. Although the effect did not reach statistical significance, there was a trend in CCTV-induced downregulation of cytokines IL-6 and IL-12p40 (though to a lesser extent), but not cytokines of the primary response (**Figure 2C**), further supporting that CCTV could have a differential effect on secondary inflammatory response (**Figure 2D**). Because IL-1β is one of the most important inflammatory molecules responsible for secondary response to LPS activation, we tested whether CCTV is able to dampen IL-1β production in LPS-stimulated BMDMs. Immunoblotting experiments demonstrated CCTV dose-dependent reduction of IL-1β in these cells, also providing a useful effective range of CCTV concentrations that was used in further experiments (**Figure 2E**). HO-1 levels were also reduced in CCTV-treated cells, however, when its concentration reached 50 µM, the effect was reversed, indicating possible over-activation of cellular antioxidant pathways.

To further characterize CCTV as a binding partner of CCR2 and determine whether this occurs concomitantly with receptor-specific activation (either agonistic or antagonistic), we performed CCL2-induced chemotaxis assays. BMDMs were pre-treated with CCTV, VTCC or vehicle followed by establishing a gradient of recombinant CCL2 in Ibidi µ-slide chemotaxis slides. The analysis of cell migration tracks over time revealed that CCTV potently inhibited CCL2-induced monocyte migration in an antagonistic manner, an effect not seen with VTCC or vehicle (**Figure 2F**). Further analysis demonstrated a statistically significant inhibition of cell velocity, distance traveled by the cells, and chemotactic index. Because CCR2 is a typical G protein-coupled receptor containing transmembrane domains, its binding to CCL2 activates actin-related signaling. In situations when such actin signalling pathways are overstimulated, a rapid induction of actin polymerization may occur that yields in cytoskeleton rearrangement, a process documented to aid in viral entry (e.g. HIV) and replication 27. Consequently, if the CCL2-CCR2 interaction is competitively disrupted by CCTV, actin polymerization may be prevented. As predicted, CCTV but not VTCC significantly inhibited actin polymerization in BMDMs overstimulated with a high dose of recombinant CCL2 (**Figure 2G**). This effect was not secondary to actin reorganization in response to endocytic engulfment of the nanoparticles, as demonstrated in BMDMs from CCR2-knockout mice that showed neither nanoparticles nor CCL2 are able to induce polymerization (**Supplementary Figure S8**).

### CCTV targets atherosclerosis and allows for imaging

We next sought to explore the ability of CCTV to interfere with a dysfunctional CCR2-CCL2 axis *In vivo.* CCL2 potentiates leukocyte chemotaxis to inflammatory sites in inflammation-central pathologies such as rheumatoid arthritis 28 and atherosclerosis 29. As previously shown by us and others^8^-^10^,^30^, molecular imaging of atherosclerosis holds the promise of identifying molecular events early on during atherogenesis allowing for the preemptive diagnosis of potentially deadly cardiovascular complications such as heart attack and stroke. Because CCTV is able to target macrophages* In vitro* (**Figure 1 and 2**), we hypothesized that CCTV could be effective for targeted imaging in macrophage-rich atherosclerotic plaques. To test this, we first determined whether *Ccl2/Ccr2* transcripts are expressed in inflammatory atherosclerotic plaque leukocytes. Using the well-validated atherosclerosis model of ApoE-knockout (ApoE^-/-^) mice fed with high-fat diet (**Figure 3A**) or control mice fed with chow; we isolated aortic leukocytes followed by gene expression analysis using nCounter Nanostring assay. nCounter uses molecular barcodes that allow for precise mRNA counts of individual transcripts even in low input samples, as few as hundreds of cells from an atherosclerotic plaque. As expected, the expression of many chemokines and their receptors changed in atherosclerotic plaque leukocytes (**Figure 3B**), with *Ccl2/Ccr2* being most significantly upregulated. Interestingly, chemokine receptors and ligand pairs known to promote leukocyte exit (egress) in atherosclerosis, such as *Ccr7/Ccl7* and *Ccr5/Ccl5*, were downregulated, consistent with prior reports 31,32 and confirming high plaque leukocyte burden in our model.

To enable imaging, the nanoparticles were supplemented with either europium (Eu) cryptates conjugated to phospholipids or DOTA-gadolinium (Gd)-conjugated lipids resulting in CCTV (VTCC)-Eu or -Gd, respectively. CCTV-Eu allowed for TRF imaging of excised organs, enabling biodistribution analysis (**Figure 3C**). Low background fluorescence and high sensitivity of TRF imaging permitted quantification of the nanoparticle signal in different organs and demonstrated that CCTV-Eu accumulated more avidly (vs. VTCC-Eu) in macrophage-rich tissues including atherosclerotic aorta, liver and lymph nodes. Conversely, VTCC-Eu accumulated prominently in liver, lung and spleen but not in the aorta, which could indicate non-specific engulfment by macrophages in these organs. MRI with CCTV-Gd revealed that these nanoparticles targeted atherosclerosis, as seen from significant delineation of the aortic wall, even 24 h after nanoparticle injection (**Figure 3D**). Further, the contrast-to-noise ratio (CNR) in CCTV-Gd-injected animals was increased compared to VTCC-Gd (**Figure 3E**). Our data suggest that CCTV selectively interacts with CCR2 and possibly other chemokine receptors in inflammatory atherosclerosis, thus allowing for delivery of imaging agents at concentrations sufficient to generate contrast enhancement, even in the low sensitivity modality of MRI.

### Cell-specific targeting via CCTV

CCTV and VTCC had similar blood clearance profiles (**Figure 4A**), however, CCTV selectively accumulated in macrophage subpopulations positive for CCR2 (**Figure 4B**) in blood of bolus nanoparticle-administered ApoE^-/-^ mice. Using a flow cytometry gating strategy that identified double-positive inflammatory CCR2^hi^Ly6C^hi^ macrophages, we demonstrated that an increased number of these cells were CCTV positive compared to VTCC. Notably, this difference was not due to a different number of macrophages that engulfed nanoparticles (**Supplementary Figure S9**). CCR2^hi^Ly6C^hi^ are responsible for chemotactic migration to the atherosclerotic plaque and are precursors to plaque burgeoning foam cells 33,34. Cross-examination of matched histopathological sections of plaque revealed that the macrophage marker F4/80 co-stained with CCTV but not VTCC rhodamine signal (**Figure 4C, D**). Our data suggest that prominent macrophage-specific accumulation of CCTV in atherosclerosis may correlate with its effects on the secondary inflammatory response (**Figure 2D**). To test this *In vivo*, ApoE^-/-^ mice were injected with a single dose of CCTV or VTCC and their atherosclerotic plaques were subjected to laser capture microdissection (LCM). LCM captured nanoparticle-positive areas of plaque (**Figure 4E**), which allowed for the expression analysis of several inflammation-related mRNAs in nanoparticle-targeted cells (**Figure 4F**). Consistent with our earlier hypothesis, CCTV downregulated levels of secondary (*Il6*, *Ccl2*) but not primary (*Tnf*, *Ifng*) response genes. Interestingly, anti-inflammatory arginase (*Arg1*) was slightly upregulated in CCTV-targeted cells, however this was not significant. Collectively, flow-cytometry and LCM data suggested CCR2-dependent cell-specific targeting by CCTV concurrent with inflammation resolution even in an acute intervention lasting just 24 h.

### CCTV perturbs NLRP3 inflammasome assembly and resolves secondary inflammatory response

**Figure 5:**
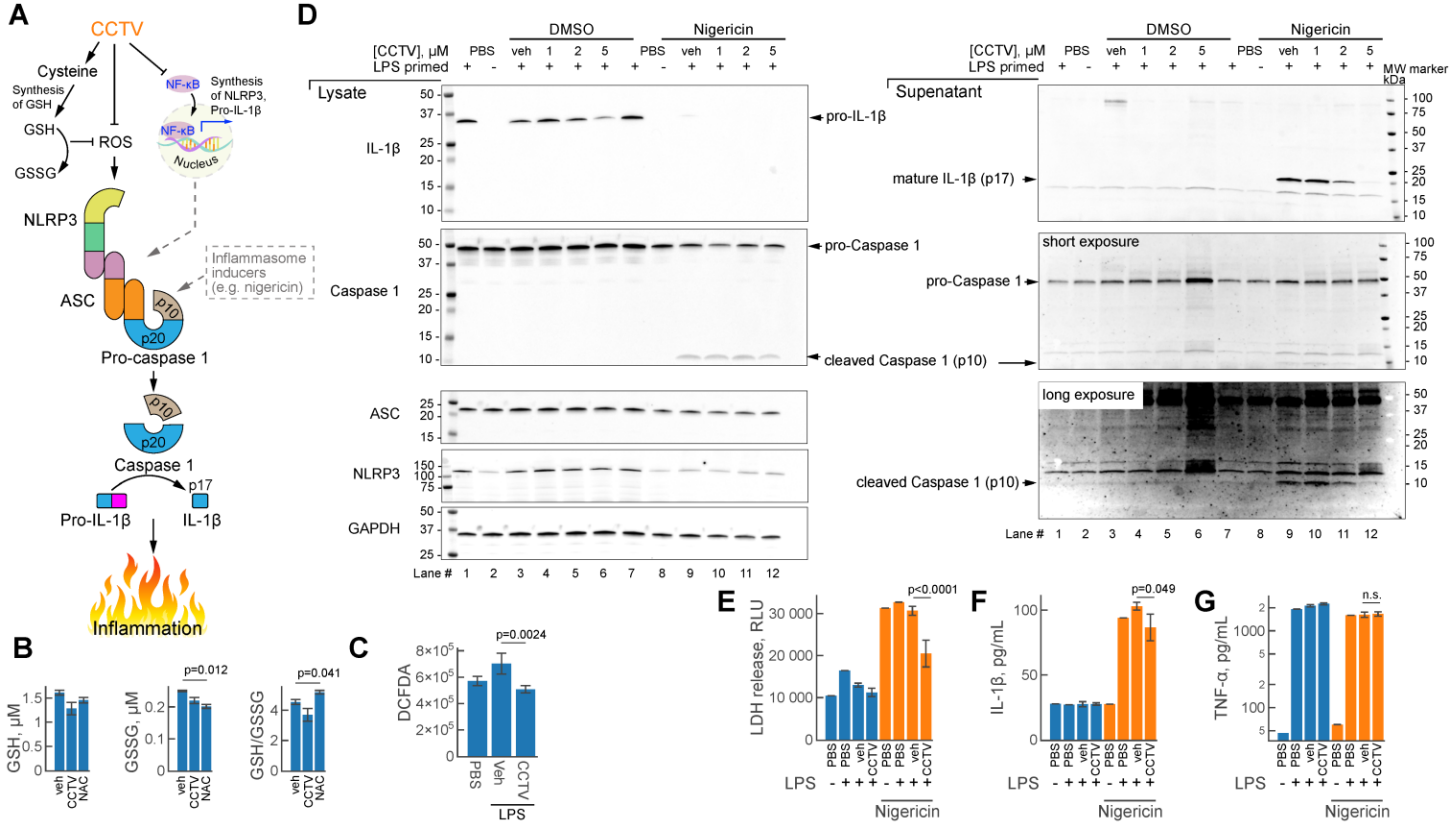
** CCTV deactivates inflammasome activation and resolves secondary inflammatory response. A)** Schematic representation of the inflammasome formation cascade and possible roles of CCTV in preventing inflammasome assembly through glutathione (GSH) and ROS (reactive oxygen species). Inflammasome (middle) is a large protein complex assembling from ASC (apoptosis-associated speck-like protein Containing CARD), NLRP3 (NLR family pyrin domain containing 3), and caspase 1 following induction (see text). Downstream inflammasome signalling involves proteolytic cleavage of pro-IL-1β releasing p17 cleaved fragment (mature IL-1β), responsible for inflammation. **B)** Oxidized (GSSG) and reduced GSH production was unaffected in lipopolysaccharide (LPS)-primed (100 ng/mL) bone marrow derived macrophages (BMDMs) treated with 5 µM CCTV as compared to vehicle (veh) but in veh vs. N-acetylcysteine (NAC). **C)** ROS generation was significantly blunted in CCTV treated, LPS stimulated (100 ng/mL) BMDMs as per 2ʹ,7ʹ-dichlorofluorescin diacetate (DCFDA) assay. **D)** Immunoblot analysis of inflammasome activation indicated CCTV dose-dependent resolution of inflammation. BMDMs were primed with 50 ng/mL LPS and treated as indicated in the presence or absence of inflammasome inducer Nigericin (5 µM) or nigericin vehicle (dimethyl sulfoxide, DMSO). Lysates and supernates were analyzed for the expression of pro- and mature-IL-1β, pro- and cleaved-caspase 1, ASC, and NLRP3. GAPDH served as a loading control. **E)** Lactate dehydrogenase (LDH) levels were determined in cell culture supernatants from the inflammasome experiments above indicating significant LDH release suppression with CCTV. **F, G)** IL-1β and TNFɑ release in cell culture supernatants as analyzed by AlphaLISA. P values are as indicated and are representative of 8 different experiments in (B), 12 in (C), 12 in (E), and 4 in (F, G).

Inflammasomes are large protein complexes that assemble intracellularly in inflammatory macrophages and other immune cells. This occurs after immune priming (e.g. pre-treatment with LPS) followed by a second “danger signal”, such as a microbial toxin from *Streptomyces hygroscopicus* nigericin, ATP or urate crystals. One important component of the inflammasome is NLRP3 (NLR family pyrin domain containing 3) encoded by *Nlrp3* mRNA and produced in typical secondary transcriptional response to inflammation (**Figure 2D**). CCTV might inhibit inflammasome assembly through NF-kB dependent regulation of the expression of inflammasome components - Nlrp3, Caspase 1 and others (**Figure 5A**), however, alternative pathways may exist. Since CCTV is enriched in cysteine amino acids, their endocytosis may give rise to elevated intracellular cysteine levels, which aids in the synthesis of glutathione (GSH). Intracellular GSH provides oxidant control (via conversion to oxidized form GSSG) in cells with elevated reactive oxygen species (ROS) during inflammation. However, this process was not observed in BMDMs pre-treated with CCTV followed by inflammation induction with LPS (**Figure 5B**). In contrast to classic antioxidant NAC, CCTV was unable to restore GSH levels or reduce GSSG in these cells. CCTV might be acting directly as ROS scavenger and thus synergistically inhibit inflammasome assembly, because ROS is known as a “secondary signal” in this process 35. Indeed, CCTV inhibited ROS production in response to LPS as seen in 2ʹ,7ʹ-dichlorofluorescin diacetate (DCFDA) assays (**Figure 5C**).

To further gain insights into the mechanism of action of CCTV on NLRP3 inflammasome, we performed a series of immunoblotting experiments in BMDMs primed with LPS followed by the treatment with prototypical inflammasome inducer nigericin (**Figure 5D**). The results show that CCTV dose-dependently inhibited the production of IL-1β and its mature form in cell culture supernatants (immunoblot lanes 9-12). The mature form of IL-1β is released via proteolytic action of active (cleaved) caspase 1, a component of the NLRP3 inflammasome (**Figure 5A**). Cleaved caspase 1 levels were also reduced in response to CCTV. Intriguingly, NLRP3 protein was markedly decreased, but only in nigericin-stimulated, CCTV-treated cells (immunoblot lanes 8-12). The findings were reproducible in J774 cells, a macrophage/monocyte cell line that was additionally tested using another inflammasome activator ATP (**Supplementary Figure S10**). These effects on NLRP3 inflammasome deactivation by CCTV were concomitant with profound rescue from nigericin-induced cell death as seen by LDH levels in cell culture supernatants (**Figure 5E**). Consistent with immunoblot data, quantification of IL-1β cytokine release in cell culture supernatants confirmed the actions of CCTV (**Figure 5F**). Strikingly, however, the levels of TNFɑ in the same supernatants were completely unaffected by CCTV treatment (**Figure 5G**). These findings and those described above confirm that CCTV is a potent anti-inflammatory nanotheranostic with effects on the secondary inflammatory response. CCTV actions in inflammatory macrophages are not confined to transcriptional regulation, but also have coordinated “re-programming” effects on the protein level, manifested by inflammasome deactivation.

## Conclusions

We serendipitously discovered CCTV, tetrapeptide-lipid self-assembled nanoparticles, while screening small library of truncated, SVM-predicted fragments of the human CCL2 protein. CCTV appears to exhibit a unique mechanism of action in inflammatory macrophages, serving both as a CCR2 antagonist and an inflammasome disruptor. We suspect that CCTV may have other mechanisms of action by which it reduces inflammation, thus warranting more detailed investigation in further studies. One important observation herein is that CCTV may have an ability to inhibit the secondary, but not the primary, inflammatory response. Our findings imply that CCTV could be efficacious in the treatment of chronic inflammation without deleterious effects on protective inflammation mediated by the primary inflammatory response. The affinity of CCTV to CCR2 in inflammatory atherosclerosis enables imaging and suggests promise for long-term benefits on plaque pathology. These observations are encouraging because recent cardiovascular clinical trials suggest that anti-inflammatory therapeutics that do not impair the immune response to infection may be beneficial.

## Supplementary Material

Supplementary figures and tables.
