# Supplementary material for: Nano-Antagonist Alleviates Inflammation and Allows for MRI of Atherosclerosis
Source: Nanotheranostics. 2019 Nov 1;3(4):342–55. doi: 10.7150/ntno.37391 (PMC6838142; doi:10.7150/ntno.37391)
Supplement: Supplementary file 1 — Supplementary figures and tables. [file ntnov03p0342s1.pdf]

Supplementary Table S1. Reagents and resources used.

Reagent or resource (abbreviation)	Obtained from (vendor)	Catalog number
DSPE-PEG(2000) Carboxylic Acid<br>(DSPE-PEG-CO2H)	Avanti Polar Lipids	880135P
DSPE-PEG(2000) Maleimide<br>(DSPE-PEG-MAL)	Avanti Polar Lipids	880135P
18:1 Liss Rhod PE<br>(Rhodamine-PE)	Avanti Polar Lipids	810150P
18:0 PE-DTPA (Gd)	Avanti Polar Lipids	791275
trFluor™ Eu-Cryptate succinimidyl ester	Aat Bioquest	1430
N-(3-Dimethylaminopropyl)-N′-ethylcarbodiimide hydrochloride<br>(EDC)	Sigma Aldrich	E6383
N-Hydroxysulfosuccinimide sodium salt<br>(sulfo-NHS)	Sigma Aldrich	56485
2-Mercaptoethanol	Sigma Aldrich	M6250
Hydroxylamine hydrochloride<br>(NH_2OH)	Sigma Aldrich	379921
Sepharose 4B	Sigma Aldrich	4B200
RAW-Blue™ Cells	Invivogen	raw-sp
Dexamethasone (Dex)	MP Biomedicals	02150154-CF
N-Acetyl-L-cysteine (NAC)	Sigma Aldrich	A9165
Nunc MaxiSorp 96-well plates	Thermo Fisher	44-2404-21
CCR2 (Human) Recombinant Protein	Abnova	H00729230-G01
Recombinant Human CCL2/MCP-1 Protein	R&D Systems	279-MC-050
Recombinant Mouse CCL2/MCP-1 Protein	R&D Systems	479-JE-010
Liberase	Roche	5401119001
Deoxyribonuclease I (Dnase I)	Roche	04536282001
Hyaluronidase	Sigma Aldrich	H3506
Lympholyte-M	Cedarlane	CL5030
RPMI-1640	Thermo Fisher	11875085
Fetal Bovine Serum (FBS)	Gemini Bio-Products	100-106
1,4-Dithiothreitol (DTT)	Roche	11583786001
Bio Rad TGX Criterion precast gels	Bio Rad	5671083

Supplementary Table S1 (continuation)		
**Reagent or resource (abbreviation)**	**Obtained from (vendor)**	**Catalog number**
AquaBlock	EastCoast Bio, Inc.	PP82
SIGMAFAST OPD	Sigma Aldrich	P9187-5SET
RAW 264.7 cells	ATCC	TIB-71
J774A.1 cells	ATCC	TIB-67
Mouse Interleukin-1ß (IL-1ß) AlphaLISA Detection Kit	Perkin Elmer	al503c
TNFα (mouse) AlphaLISA Detection Kit	Perkin Elmer	al505c
Streptavidin-HRP	Thermo Fisher	21132
anti-NLRP3/NALP3, used at 1:5000 for Western blot	Adipogen	AG-20B-0014-C100
anti-Caspase-1 (p10), used at 1:500 for Western blot	Adipogen	AG-20B-0044-C100
Biotin anti-human MCP-1 Antibody	BioLegend, Inc.	502609
Anti-F4/80 antibody [CI:A3-1] (ab6640), used at 1:250 for IHC	Abcam	ab6640
Cleaved Caspase-1, used at 1:1000 for Western blot	Cell Signalling	67314S
GAPDH-HRP, used at 1:2000 for Western blot	Cell Signalling	8884S
IL-1-Beta, used at 1:5000 for Western blot	Cell Signalling	31202S
Caspase-3, used at 1:1000 for Western blot	Cell Signalling	14220S
HO-1, used at 1:10,000 for Western blot	Cell Signalling	82206S
ASC/TMS1, used at 1:1000 for Western blot	Cell Signalling	67824T
beta Actin Polyclonal Antibody, used at 1:30,000 for Western blot	Thermo Fisher	PA1-16889
CD11b, used at 1 μL per 10^6 cells in flow cytometry	BioLegend, Inc.	101211
CD11c used at 1 μL per 10^6 cells in flow cytometry	BioLegend, Inc.	117321
CCR2 used at 1 μL per 10^6 cells in flow cytometry	BioLegend, Inc.	150609
Ly6C used at 1 μL per 10^6 cells in flow cytometry	BioLegend, Inc.	128021
Goat anti-Mouse IgG (H+L), Superclonal Recombinant Secondary Antibody, HRP, at 1:50,000 for Western blot	Thermo Fisher	A28177
Goat anti-Rabbit IgG (H+L), Superclonal™ Recombinant Secondary Antibody, HRP, at 1:50,000 for Western blot	Thermo FIsher	A27036
μ-Slide Chemotaxis	IBIDI	80326
ibidi Heating System	IBIDI	10918

Supplementary Table S1 (continuation)		
**Reagent or resource (abbreviation)**	**Obtained from (vendor)**	**Catalog number**
Alexa Fluor 633 Phalloidin	Thermo FIsher	A22284
Qiagen RNeasy Mini Kit	Qiagen	74104
High-Capacity cDNA Reverse Transcription Kit	Thermo FIsher	4368814
Mrc1 TaqMan	Thermo FIsher	Mm01329632_m1
Nos2 TaqMan	Thermo FIsher	Mm00440502_m1
IL6 TaqMan	Thermo FIsher	Mm00446190_m1
TNF TaqMan	Thermo FIsher	Mm00443258_m1
IL1b TaqMan	Thermo FIsher	Mm00434228_m1
Teklad Custom Diet	ENVIGO	TD.02028
Alfalfa-free diet	ENVIGO	TD.97184
DAB Peroxidase (HRP) Substrate Kit	Vactor Labs	SK-4100
Nigericin	Invivogen	tlrl-nig
Adenosine 5′-triphosphate (ATP)	Sigma Aldrich	A1852
nCounter Inflammation Panel	Nanostring	XT-CSO-MIN2-12
Mouse Cytokine Array - Eve Technologies	EVE technologies	MD31
GSH/GSSG-Glo Assay	Promega	V6611
DCFDA / H2DCFDA - Cellular ROS Assay Kit	Abcam	ab113851
LDH-Glo Cytotoxicity Assay	Promega	J2380
B6.129P2-Apoe^{tm1Unc}/J mice	The Jackson Laboratory	Stock No: 002052
B6.129S4-Ccr2^{tm1Ifc}/J mice	The Jackson Laboratory	Stock No: 004999
BMS CCR2 22	Tocris	Cat. No. 3129
ULTRA PURE LPS from *Salmonella minnesota* R595 (Re)	List Biological Laboratories	434
ACK lysis buffer	Thermo FIsher	A1049201

Supplementary Table S2. Peptides Scanned from Original Protein.
MKVSAALLCLLLIAATFIPQGLAQPDAINAPVTCCYNFTNRKISVQRLASYRRITSSKCPKEAVIFKTIVAKEICADPKQKWVQDSMDHLDKQTQTPKT

Peptide Sequence	SVM score	Prediction	Hydrophobicity	Hydropathicity	Hydrophilicity	Charge	Mol wt
RLAS	-0.77	AHT	-0.31	0.07	0.25	1	445.55
KVSA	-0.74	AHT	-0.14	0.33	0.33	1	403.52
LLIA	-0.71	AHT	0.51	3.48	-1.48	0	428.6
KISV	-0.65	AHT	-0.02	1	0	1	445.6
**VTCC**	**-0.64**	**AHT**	**0.11**	**2.12**	**-0.97**	**0**	**424.57**
KTIV	-0.64	AHT	0	1.03	-0.17	1	459.63
DKQT	-0.64	AHT	-0.67	-2.9	1.45	0	490.56
IVAK	-0.62	AHT	0.1	1.65	-0.2	1	429.6
HLDK	-0.62	AHT	-0.42	-1.7	0.93	0	511.62
ALLC	-0.56	AHT	0.34	2.97	-1.27	0	418.58
QDSM	-0.55	AHT	-0.35	-1.48	0.55	-1	479.55
LLLI	-0.54	AHT	0.58	3.97	-1.8	0	470.68
MKVS	-0.53	AHT	-0.14	0.35	0.12	1	463.64
MDHL	-0.53	AHT	-0.08	-0.25	-0.15	-1	514.64

Supplementary Table S3. Physico-chemical properties of synthesized nanoparticles.

NP ID	Conjugated Peptide	Conjugation end N-Term or SH type	Size (Mean Z average), nm	Size Standard Deviation	Mean Polydispersity Index (PDI)
A1	RLAS	N	93	11	0.198
A2	KVSA	N	110	32	0.124
A3	LLIA	N	134	42	0.162
A4	KISV	N	114	42	0.09
A5	VTCC	N	76	10	0.141
A6	KTIV	N	142	32	0.054
B1	DKQT	N	204	54	0.176
B2	IVAK	N	53	5	0.058
B3	HLDK	N	61	12	0.257
B4	ALLC	N	86	14	0.151
B5	QDSM	N	198	65	0.169
B6	LLLI	N	223	32	0.152
C1	MKVS	N	189	23	0.159
C2	MDHL	N	134	13	0.111
C3	VTCC	SH	167	64	0.148
C4	ALLC	SH	79	23	0.002

Supplementary Table S4. Alignment of CCTV filtered by *mus musculus*.

Sequence ID	Start	End	Organism	Protein
NP_038658.2	3,273	3,276	*Mus musculus*	polycystin-1 precursor
NP_076055.3	1,533	1,536	*Mus musculus*	mucin-2 precursor
NP_001240786.1	1,641	1,644	*Mus musculus*	methylcytosine dioxygenase TET1 isoform 1
NP_082290.2	1,000	1,003	*Mus musculus*	transmembrane protein 94
NP_001116066.2	978	981	*Mus musculus*	PH domain leucine-rich repeat-containing protein phosphatase 2
NP_001076811.1	553	556	*Mus musculus*	protein dispatched homolog 3
NP_001074813.2	400	403	*Mus musculus*	syntaxin-binding protein 5
NP_780465.4	971	974	*Mus musculus*	protein HEG homolog 1 precursor
NP_666151.3	642	645	*Mus musculus*	WD repeat-containing protein 60
NP_899006.1	174	177	*Mus musculus*	G protein-regulated inducer of neurite outgrowth 3
NP_941052.1	340	343	*Mus musculus*	solute carrier family 22 member 20
NP_032568.3	123	126	*Mus musculus*	mothers against decapentaplegic homolog 6
NP_033332.1	72	75	*Mus musculus*	synaptotagmin-1 isoform 1
NP_001239271.1	72	75	*Mus musculus*	synaptotagmin-1 isoform 2
NP_032132.1	220	223	*Mus musculus*	medium-wave-sensitive opsin 1
NP_796007.1	18	21	*Mus musculus*	brorin precursor
NP_666413.1	143	146	*Mus musculus*	olfactory receptor 1475
NP_997015.2	143	146	*Mus musculus*	olfactory receptor 1471
NP_666901.2	143	146	*Mus musculus*	olfactory receptor 1472
NP_071297.1	148	151	*Mus musculus*	fructosamine-3-kinase isoform a
NP_852085.2	148	151	*Mus musculus*	ketosamine-3-kinase
NP_001033788.1	148	151	*Mus musculus*	fructosamine-3-kinase isoform b
NP_061262.1	94	97	*Mus musculus*	spermatid-specific linker histone H1-like protein
NP_035461.2	31	34	*Mus musculus*	**C-C motif chemokine 12 precursor**
NP_067418.1	30	33	*Mus musculus*	**C-C motif chemokine 8 precursor**

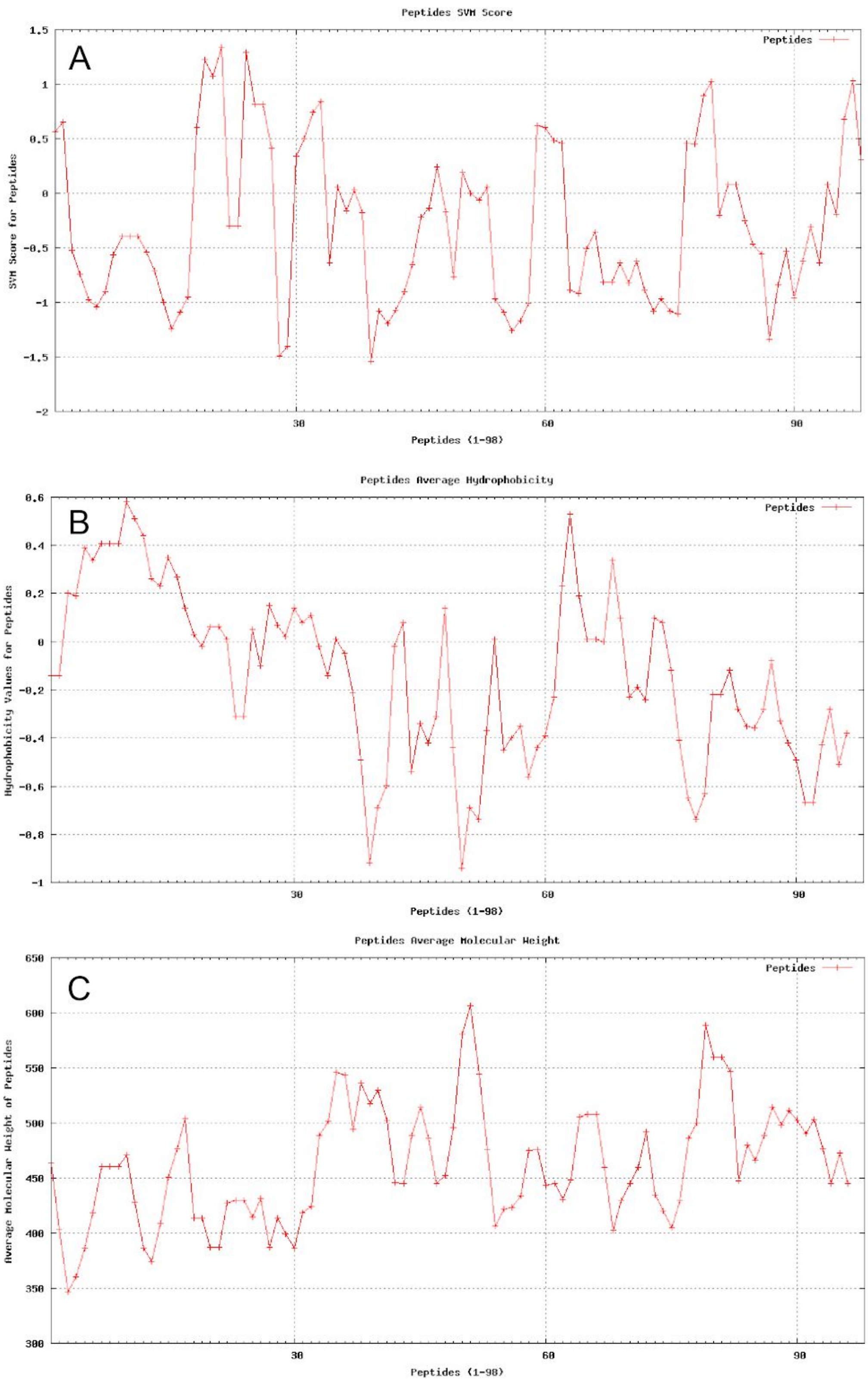

Supplementary Figure S1. Peptides and their characteristics predicted via Support Vector Machine (SVM) and the publicly available tool http://crdd.osdd.net/raghava/ahtpin. A) SVM scores for predicted peptides, B) Average hydrophobicity of predicted peptides, C) Average molecular weight of predicted peptides.

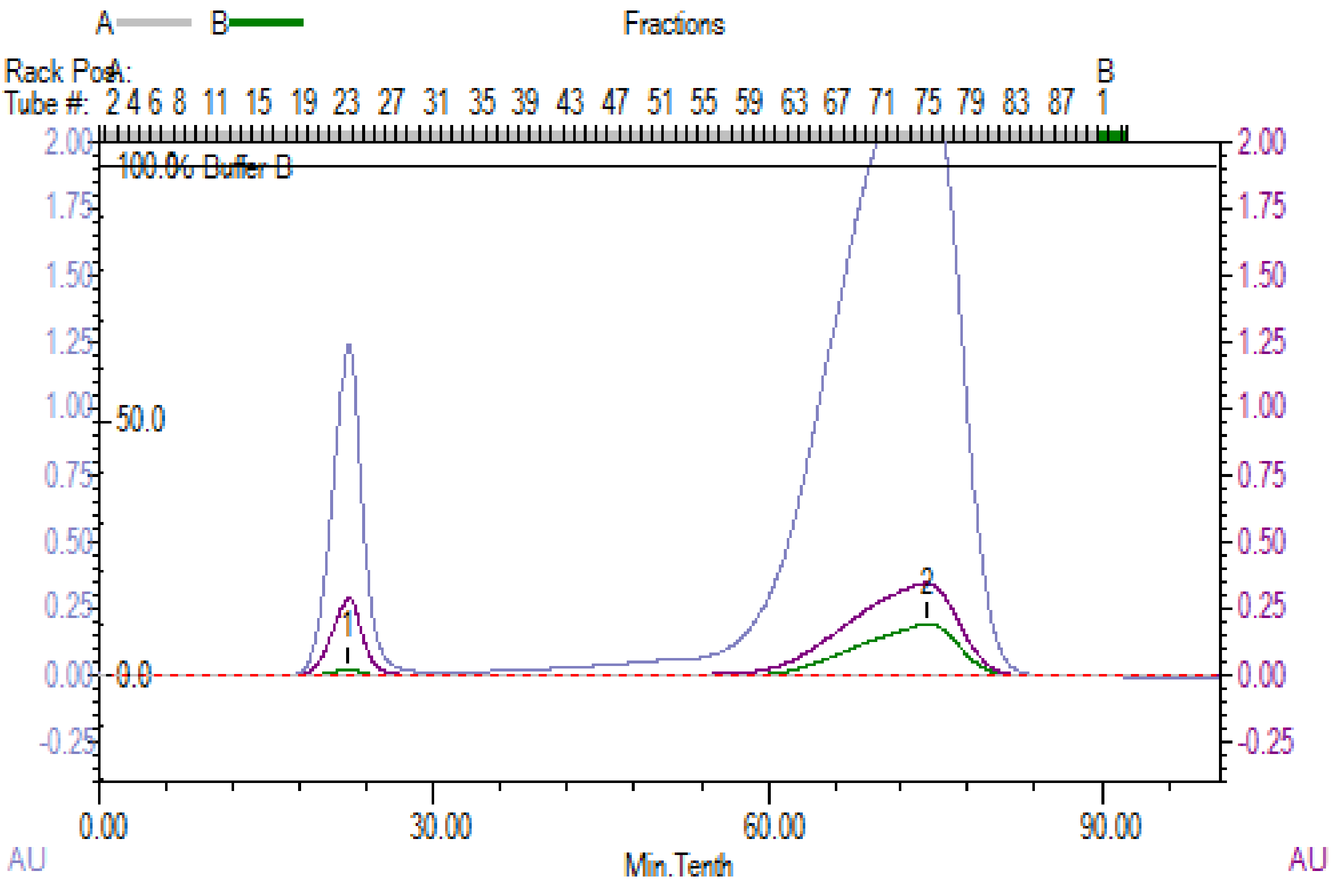

Supplementary Figure S2. Gel filtration purification of obtained nanoparticles. Fractions 15-31 (peak 1) were collected for further processing. Chromatogram traces of different colors represent different UV wavelengths of the detector.

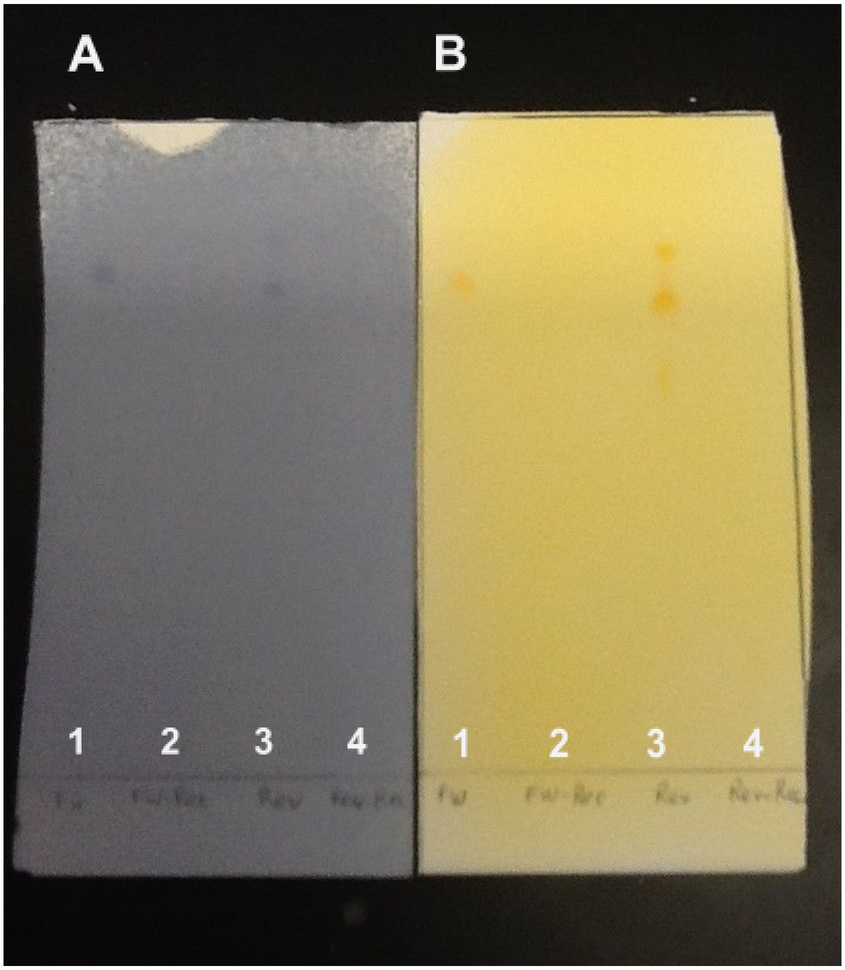

Supplementary Figure S3. TLC of obtained conjugation products. A) Molybdenum blue staining phosphates; B) Dragendorff reagent staining amines. 1: N-terminus CCTV conjugation; 2: reaction residue; 3: CCTV SH-conjugation; 4: reaction residue.

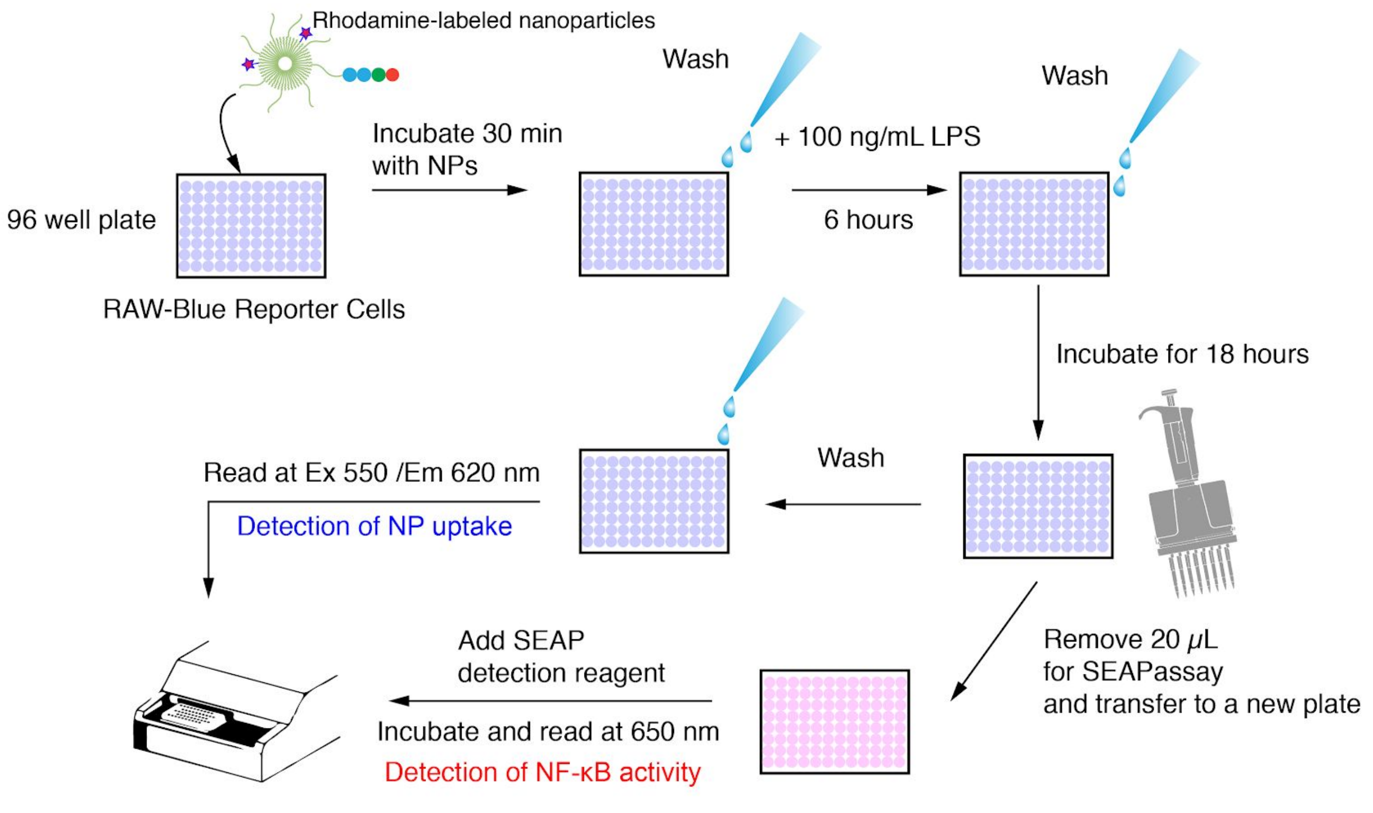

Supplementary Figure S4. Schematic representation of screening.

Mouse alignment

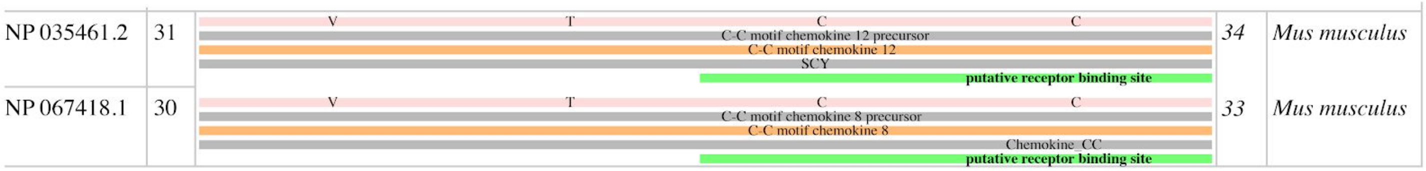

Human alignment

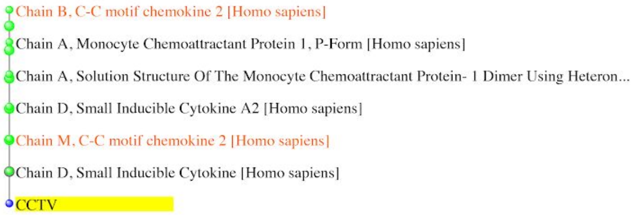


```
>pdb|4ZK9|B
-QPDAINAPVTCCYNFTNRKISVQRLASYRRITSSKCPKEAVIFKTIVAKEICADPKQKW
VQDSMDHLDKQTQTPKTHHHHHHH
>pdb|1DOK|A
MQPDAINAPVTCCYNFTNRKISVQRLASYRRITSSKCPKEAVIFKTIVAKEICADPKQKW
VQDSMDHLDKQTQTPKT-------
>pdb|1DOM|A
-QPDAINAPVTCCYNFTNRKISVQRLASYRRITSSKCPKEAVIFKTIVAKEICADPKQKW
VQDSMDHLDKQTQTPKT-------
>pdb|2NZ1|D
-QPDAINAPVTCCYNFTNRKISVQRLASYRRITSSKCPKEAVIFKTIVAKEICADPKQKW
VQDSIDHLDKQTQTPKT-------
>pdb|4DN4|M
-QPDAINAAVTCCYNFTNRKISVQRLASYRRITSSKCPKEAVIFKTIVAKEICADPKQKW
VQDSMDHLDKQTQTPKT-------
>pdb|1ML0|D
-QPDAINAAVTCCYNFTNRKISVQRLASYRRITSSKCPKEAVIFKTIVAKEICADPKQKW
VQDSIDHLDKQTQTPKT-------
```

Supplementary Figure S5. FASTA alignment of the CCTV sequence using a constraint-based multiple alignment tool (https://www.ncbi.nlm.nih.gov/tools/cobalt/) demonstrated alignment of CCTV with putative receptor binding sites in CC motif chemokine 12 and 8 in mouse sequences and with CC motif chemokine 2 in human sequences.

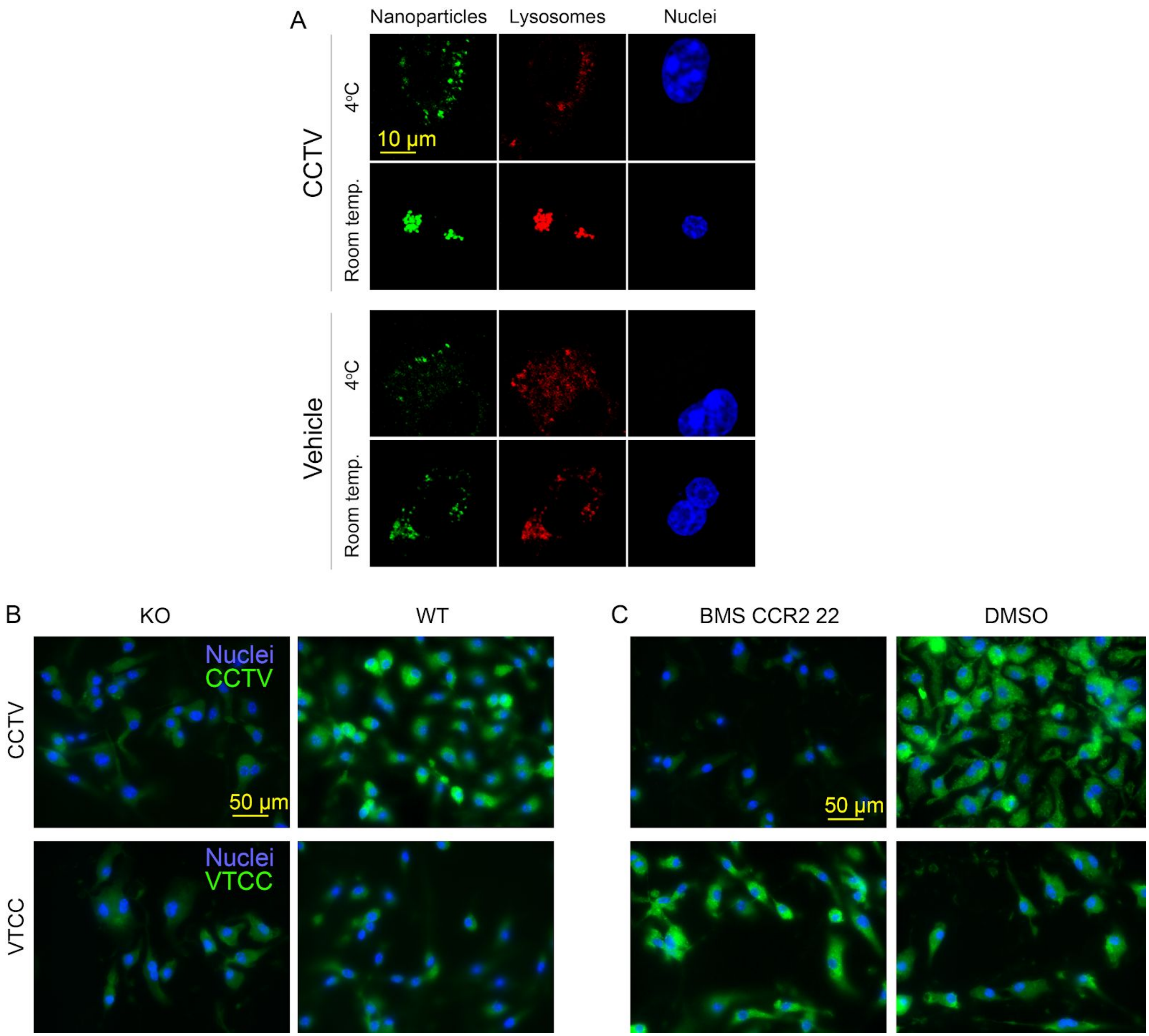

Supplementary Figure S6. A) Separate channels for main manuscript Figure 1B.
Uptake of carboxyfluorescein-labeled CCTV or VTCC in cultured bone marrow derived macrophages from **B)** CCR2 knockout (KO, B6.129S4-Ccr2^{tm1Ifc}/J) or wild-type (WT) mice; **C)** wild-type mouse-derived macrophages pre-treated (6 h) with CCR2 inhibitor BMS CCR2 22 (10 nM) or DMSO as vehicle control. **C)**

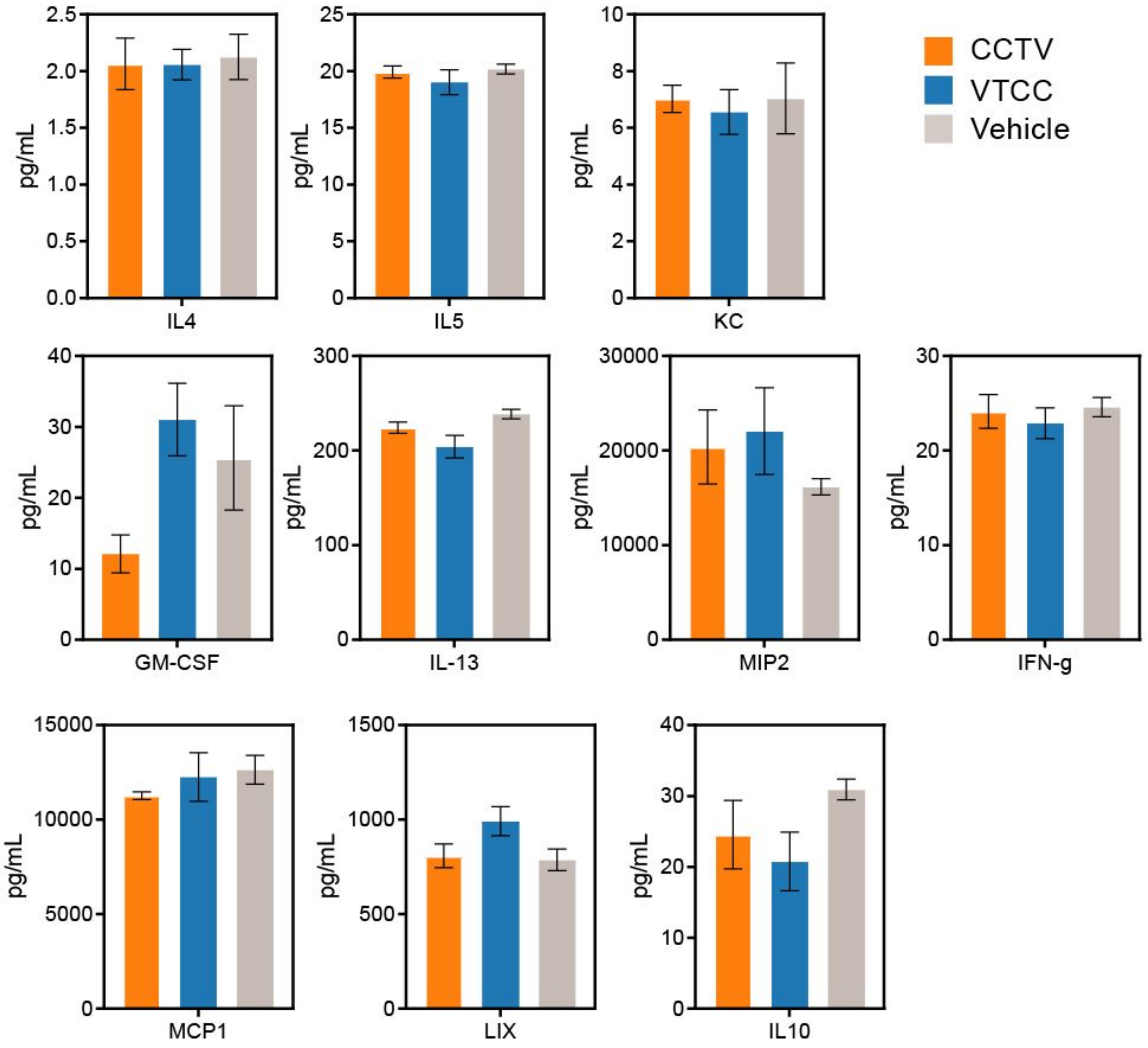

Supplementary Figure S7. Cytokines in supernatants from RAW cells stimulated with lipopolysaccharide (100 ng/mL) and treated with CCTV, VTCC or vehicle nanoparticles.

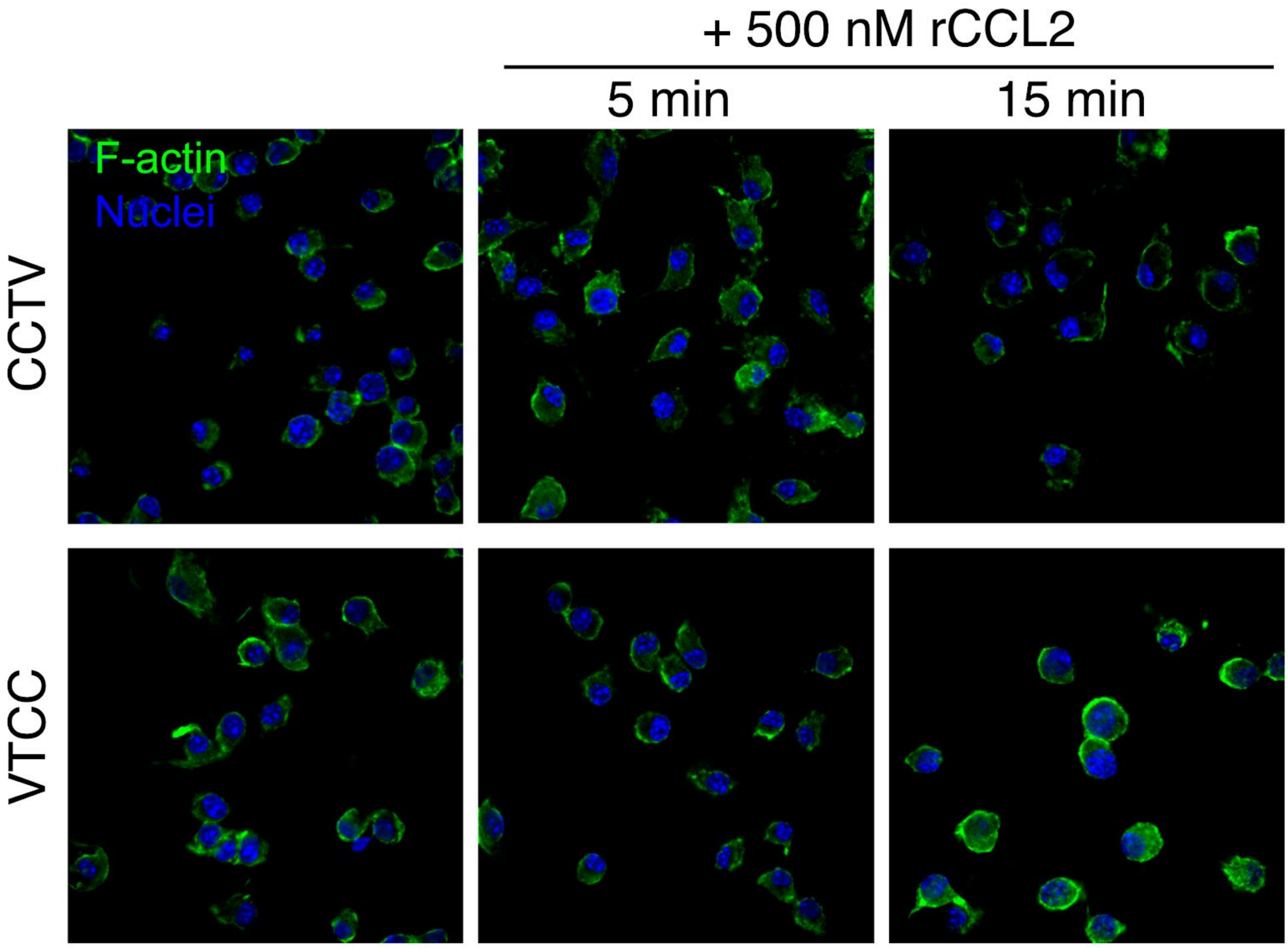

Supplementary Figure S8. Actin polymerization assays in bone marrow derived monocytes isolated from CCR2 knockout mice (B6.129S4-Ccr2^{tm1Ifc}/J). The experiments were performed as described in Methods and main text. Green: phalloidin staining of actin, blue: nuclei stained with Hoechst 33342.

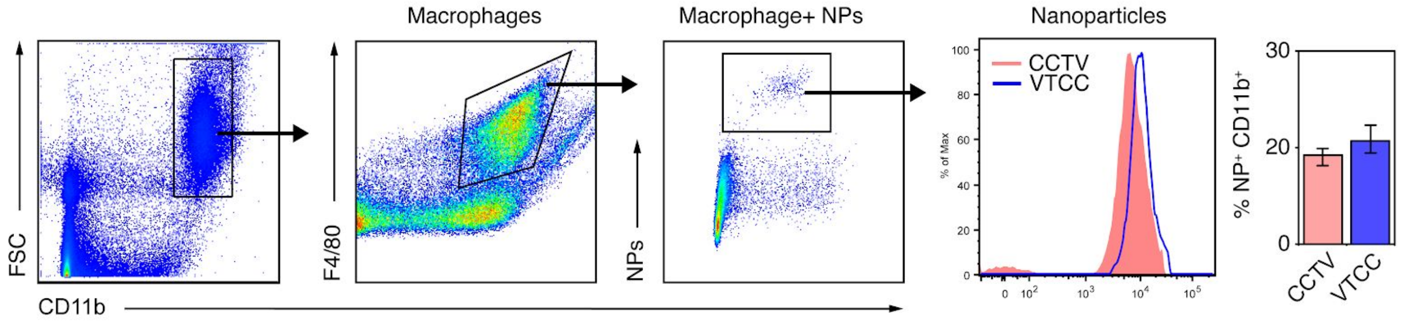

Supplementary Figure S9. Flow cytometry analysis of total macrophage-specific accumulation of CCTV or VTCC in blood of Apoe$^{-/-}$ nanoparticle injected mice. The percent of macrophages that engulfed either nanoparticle was the same. Total blood macrophages were identified as F4/80 positive and CD11b positive.

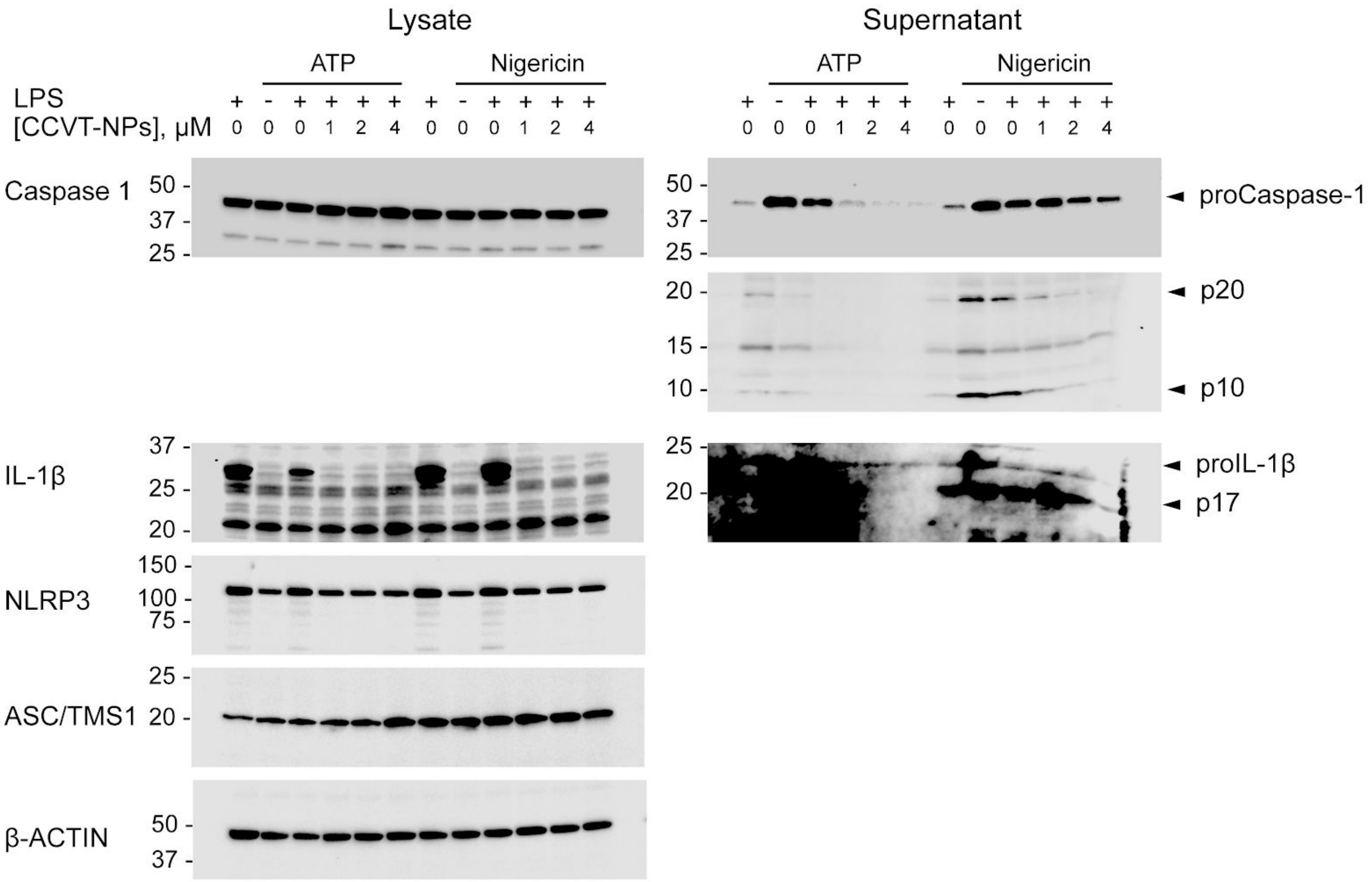

Supplementary Figure S10. Inflammasome activation assays in J774 cells. The cells were treated as described in Methods and main text Figure 5 legend, plus additional inflammasome activator ATP that was added at 1 mM. Immunoblot was performed as described in methods.
